# Etiologic effects and optimal intakes of foods and nutrients for risk of cardiovascular diseases and diabetes: Systematic reviews and meta-analyses from the Nutrition and Chronic Diseases Expert Group (NutriCoDE)

**DOI:** 10.1371/journal.pone.0175149

**Published:** 2017-04-27

**Authors:** Renata Micha, Masha L. Shulkin, Jose L. Peñalvo, Shahab Khatibzadeh, Gitanjali M. Singh, Mayuree Rao, Saman Fahimi, John Powles, Dariush Mozaffarian

**Affiliations:** 1 Friedman School of Nutrition Science and Policy, Tufts University, Boston, Massachusetts, United States of America; 2 University of Michigan Medical School, Michigan, Ann Arbor, Michigan, United States of America; 3 Brandeis University, Heller School for Social Policy and Management, Waltham, Massachusetts, United States of America; 4 The Warren Alpert Medical School of Brown University, Providence, Rhode Island, United States of America; 5 Digestive Disease Research Institute, Tehran University of Medical Sciences, Tehran, Iran; 6 Cambridge Institute of Public Health, Cambridge, United Kingdom; Medizinische Universitat Innsbruck, AUSTRIA

## Abstract

**Background:**

Dietary habits are major contributors to coronary heart disease, stroke, and diabetes. However, comprehensive evaluation of etiologic effects of dietary factors on cardiometabolic outcomes, their quantitative effects, and corresponding optimal intakes are not well-established.

**Objective:**

To systematically review the evidence for effects of dietary factors on cardiometabolic diseases, including comprehensively assess evidence for causality; estimate magnitudes of etiologic effects; evaluate heterogeneity and potential for bias in these etiologic effects; and determine optimal population intake levels.

**Methods:**

We utilized Bradford-Hill criteria to assess probable or convincing evidence for causal effects of multiple diet-cardiometabolic disease relationships. Etiologic effects were quantified from published or *de novo* meta-analyses of prospective studies or randomized clinical trials, incorporating standardized units, dose-response estimates, and heterogeneity by age and other characteristics. Potential for bias was assessed in validity analyses. Optimal intakes were determined by levels associated with lowest disease risk.

**Results:**

We identified 10 foods and 7 nutrients with evidence for causal cardiometabolic effects, including protective effects of fruits, vegetables, beans/legumes, nuts/seeds, whole grains, fish, yogurt, fiber, seafood omega-3s, polyunsaturated fats, and potassium; and harms of unprocessed red meats, processed meats, sugar-sweetened beverages, glycemic load, *trans*-fats, and sodium. Proportional etiologic effects declined with age, but did not generally vary by sex. Established optimal population intakes were generally consistent with observed national intakes and major dietary guidelines. In validity analyses, the identified effects of individual dietary components were similar to quantified effects of dietary patterns on cardiovascular risk factors and hard endpoints.

**Conclusions:**

These novel findings provide a comprehensive summary of causal evidence, quantitative etiologic effects, heterogeneity, and optimal intakes of major dietary factors for cardiometabolic diseases, informing disease impact estimation and policy planning and priorities.

## Introduction

Cardiometabolic diseases including coronary heart disease (CHD), stroke, and type 2 diabetes are leading causes of morbidity and mortality globally [1]. In 2011, the United Nations highlighted suboptimal diet as one of the principal drivers of these diseases [2]. Our collaborative work in the Global Burden of Diseases (GBD) Study demonstrated that 8 of the top 20 risk factors for lost disability-adjusted life-years globally were dietary factors; and that several more of the top 20 were strongly diet-related, including high blood pressure, body mass index (BMI), fasting plasma glucose, and total cholesterol [3]. In sum, suboptimal diet is one of the leading preventable causes of death and disability in the US and globally [3–6].

To determine the impact of specific dietary factors on cardiometabolic diseases and inform priorities for intervention and prevention, it is crucial to understand the strength of evidence on causality, the magnitudes of disease-specific etiologic effects (e.g., relative risks [RRs]), the heterogeneity in these effects by underlying individual characteristics such as age or sex, and the optimal levels of consumption for reducing risk. Yet, these key questions have not previously been systematically assessed nor comparably reviewed for CHD, stroke, and diabetes. Though some evidence on diet and cardiometabolic diseases has been previously assessed, no contemporary investigation comprehensively evaluated multiple dietary factors while also including qualitative assessment of evidence for causality [7], quantitative assessments of etiologic dose-responses [7] and optimal consumption levels [7–9].

To address these gaps in knowledge, we systematically reviewed the evidence for effects of dietary factors on cardiometabolic diseases, including comprehensively assess evidence for causality; estimate magnitudes of etiologic effects focusing on dose-responses rather than simple categorical comparisons; evaluate heterogeneity and potential for bias in these etiologic effects; and determine optimal population intake levels. We hypothesized that certain individual dietary components would have probable or convincing evidence for causal effects on cardiometabolic diseases; and that magnitudes of estimates would be reasonably unbiased based on validity analyses.

## Methods

### Evidence for causality

Our methods for evaluating strength of evidence for causal diet-chronic disease relationships were reported [10]. We searched for dietary factors with evidence for causal effects on total cardiovascular disease (CVD), CHD, stroke, or diabetes. Given paucity of evidence from randomized controlled trials, our primary determinations were based on Bradford-Hill criteria [11] graded independently and in duplicate (RM, DM), including evidence on strength/consistency, temporality, coherence, specificity, analogy, plausibility, biological gradient, and supportive experimental data (Text A in S1 File). In our final analysis, we conservatively only included factors which were determined to have probable or convincing evidence for causal effects. Based on our and other recent reviews [12], many dietary factors were evaluated and determined not to achieve these criteria for causality; e.g., a leading candidate not achieving sufficient evidence was coffee, and others were extra-virgin olive oil, monounsaturated fat, cocoa, and tea (Text B in S1 File). We also qualitatively considered concordance of our conclusions with other criteria for causality of diet-chronic disease relationships as probable or convincing, such as from the World Health Organization (WHO) and WCRF/AICR [13–15]. Overall, we elected to be conservative in our approach, excluding rather than including dietary factors with borderline judgments on at least probable causal evidence. As evidence continues to accrue, we hope to update this investigation in future years using similar standardized methods. For the present work focused on diet, we did not evaluate alcohol which is often considered separately as a potentially addictive substance, is implicated in accidental deaths, and for which health effects have been evaluated [16].

### Literature searches for etiologic effects

For each identified diet-disease relationship, we performed multiple systematic searches of PubMed through 1/May/2015 to identify meta-analyses of randomized controlled trials (RCTs) or prospective cohort studies evaluating these specific dietary factors and total CVD, CHD including subtypes (fatal, nonfatal), stroke including subtypes (ischemic, hemorrhagic), or diabetes. For sodium and sugar-sweetened beverages (SSBs), we also reviewed effects on blood pressure (BP) and obesity, respectively, based on RCTs demonstrating primary effects on these risk pathways. Our detailed protocol for identifying studies on etiologic effects of dietary habits on chronic diseases has been reported [10]. Search terms and results are provided in S1 File (Text B, Table A, Figure A). For each search, one investigator screened all electronically identified titles and abstracts and, for all articles selected for full-text review, further hand-searched the citation lists and also the first 20 “related articles” in PubMed. These searches were supplemented with additional expert contacts to identify all potentially relevant articles.

For a few dietary factors for which evidence for causal effects on specific cardiometabolic outcomes was identified, recently published meta-analyses were unavailable. For these diet-disease relationships, we performed *de novo* meta-analyses according to PRISMA guidelines (S2 File) [17]. These included systematic searches of online databases and hand-searching of reference lists and related citations. For each meta-analysis, titles and abstracts of identified studies were screened by one investigator, and relevant full-texts were reviewed independently and in duplicate by two investigators. Protocols for these meta-analyses are provided in S1 File (Text C-D).

### Study inclusion

Published meta-analyses were eligible if including RCTs or prospective cohorts of the identified diet-disease relationship of interest. Whenever possible, we prioritized meta-analyses that characterized dose-responses using all available data (as opposed to comparisons of extreme categories, e.g., high vs. low). Meta-analyses including only retrospective case-control studies were excluded due to greater potential for selection bias, recall bias, and reverse causation. When more than one meta-analysis was identified for any diet-disease relationship, we included the dose-response analysis with the greatest number of studies and clinical events. When recent meta-analyses were identified, they were not updated. We only included published, peer-reviewed meta-analyses; or performed *de novo* meta-analyses with all methods presented. For new meta-analyses, we included all RCTs and prospective cohorts that assessed the diet-disease relationship of the interest (Text C in S1 File). Studies were excluded if they only reported crude estimates, lasted less than 3 months, or focused on special populations (e.g., comparisons of vegetarians vs. non-vegetarians).

### Data extraction

For each published meta-analysis, we extracted data independently and in duplicate using a standardized electronic spreadsheet on definitions of dietary factors and outcomes, numbers of studies included, pooled risk estimates and corresponding uncertainty, study designs, sample sizes, numbers of events, mean ages of participants at baseline, lengths of follow-up, ranges of intake, statistical methods, evidence for bias, and control for confounders in individual studies. In most cases, all required data were not reported in the original meta-analyses and were therefore extracted from the original individual studies cited in the meta-analysis.

For new meta-analyses, data were extracted independently and in duplicate from each identified individual study using a standardized electronic spreadsheet. Data were extracted on author name, contact information, publication year, study name, location, design, population (age, sex, race, sample size), follow-up duration, exposure/intervention (definition, assessment, categories, dose in each category), outcome (definition, ascertainment), analysis method, covariates, number of events, and the risk estimate and its corresponding uncertainty in each exposure/intervention category. For each meta-analysis, we standardized the risk estimates to a common standardized serving size to enable comparability across studies.

### Evidence synthesis

Data synthesis utilized published results when dose-response meta-analyses were performed in the published report, or categorical comparisons when such findings were unavailable; and otherwise, when possible, data from the individual original articles in each meta-analysis to perform new dose-response meta-analyses. For *de novo* meta-analyses, we extracted data on each individual study as described above and performed random-effects two-step generalized least squares for trend estimation (GLST command in STATA) [18, 19]. This method utilizes all available data to compute study-specific dose-response estimates based on the natural log relative risk (RR) in each category of intake and pools these to estimate an overall RR for a standardized serving and frequency of intake. We assessed between-study heterogeneity using Cochran's Q and I^2^ statistic. I^2^ values of 25–50%, 50–75%, and >75% were considered to represent low, moderate, and high heterogeneity, respectively [20]. Potential for publication bias was explored statistically using Begg’s test [21] and by visual inspection of funnel plots. All analyses were conducted with STATA 14.0 software (StataCorp).

### Heterogeneity in etiologic effects

Proportional effects (RRs) of major cardiometabolic risk factors have been shown to decline with age [3, 22]. To quantify and incorporate this effect modification by age, we evaluated the proportional differences in RRs for major diet-related cardiometabolic risk factors, including systolic blood pressure (SBP), body mass index (BMI), fasting plasma glucose and total cholesterol, across 6 age groups from 25–34 to 75+ years (Text F & Figure B in S1 File) [22–25]. Because proportional differences between adjacent age groups were quite similar across these four risk factors, we applied the mean proportional differences in RR by age across all risk factors to the dietary RRs, anchored at the mean age at event of each diet-disease pair. In applying these to diet, we used Monte Carlo simulations to estimate the uncertainty in the age-distributed log RRs, sampling from the distribution of log RRs at the age at event. Based on 1,000 simulations, we utilized the 2.5^th^ and 97.5^th^ percentiles to derive the 95% uncertainty interval, hereafter described as the 95% confidence interval (CI). We also reviewed the findings of meta-analyses to consider potential effect modification by sex and, where relevant, other factors such as race, hypertensive status, and BMI.

### Optimal intakes

To permit comparable quantitative assessment of impacts on disease, we characterized the optimal population consumption levels of each dietary factor for risk of cardiometabolic diseases [10, 26]. Optimal levels were selected primarily based on risk (observed consumption levels associated with lowest disease risk in meta-analyses) with further considerations of feasibility (observed national mean consumption levels in nationally representative surveys worldwide) [27–32] and consistency with other assessments (existing major dietary guideline reports) [33–35]. Because populations inevitably have a range of consumption levels, we utilized a normal distribution around each optimal intake level with standard deviation (SD) equaling 10% of the mean, consistent with optimal distribution ranges of metabolic risk factors [3, 36–39].

### Assessment of validity and bias

Estimated etiologic effects could be limited by confounding (typically causing overestimation of effects) and measurement error (typically causing underestimation of effects). Measurement error was generally not addressed in most studies, although some utilized serial measures of diet. To reduce bias from confounding nearly all identified observational studies evaluating etiologic effects utilized multivariable adjustment for major demographic factors and, in many cases, other dietary factors. Yet, we recognized that clustering of dietary patterns could still cause unmeasured confounding, e.g., from clustering of healthful factors such as fruits, vegetables, and whole grains and inverse correlations of these with harmful factors such as SSBs or processed meats. Thus, even with multi-variable adjustment, our final calculated etiologic effects from studies of an individual dietary component might overestimate its effects, as compared with the true effect when the dietary component is consumed as part of an overall diet pattern.

To assess potential bias from dietary pattern effects, we performed 3 validity analyses (Tables S4-S6 in S1 File), based on: (a) prospective long-term observational studies evaluating overall dietary patterns and clinical cardiovascular events; (b) randomized controlled feeding trials evaluating overall dietary patterns and cardiovascular risk factors (LDL-cholesterol, SBP); and (c) a large RCT evaluating overall dietary patterns and clinical cardiovascular events. For each, we compared the observed effect from the dietary pattern study to the estimated RR calculated by jointly considering the individual etiologic effects (RRs) for each dietary component in that pattern.

For prospective cohorts evaluating overall diet patterns and CVD events [40–44], the observed multivariable-adjusted RR in each category (e.g., quintile) of the dietary pattern was compared to the estimated effect calculated by combining the reported differences in each individual dietary component (e.g., fruits, nuts) across each category of the diet pattern with our estimated individual etiologic effect (RR) for that dietary component, assuming a multiplicative relation between RRs for individual components. We focused on foods and excluded overlapping components (e.g., we included whole grains, fruits, and vegetables; and excluded dietary fiber); we also assumed no benefits from differences in other dietary factors (e.g., coffee) in the pattern for which we had not determined a causal etiologic effect.

For randomized controlled feeding trials of dietary patterns and CVD risk factors, we performed inverse-variance-weighted meta-regression across all of the treatment arms of three large, well-established dietary pattern trials [45–47] to estimate the independent effects of five individual dietary components, when consumed as part of an overall dietary pattern, on SBP and LDL-cholesterol. We evaluated achieved changes in fruits, vegetables, nuts, whole grains, and fish simultaneously as independent variables, with changes in SBP or LDL-C as the dependent variable. For each dietary component, we then calculated how the identified change in SBP and LDL-C from the meta-regression would alter cardiovascular risk, based on the established relationship between SBP and LDL-C and clinical events [48–52] assuming independent, multiplicative effects of SBP and LDL-C. These effects, calculated based only on how each dietary component altered SBP and LDL-C in randomized controlled feeding trials of diet patterns, were then compared to our estimated etiologic effect on cardiovascular events for that dietary component. We recognized that the calculated effects based on the feeding trial results might be conservative, as they presume that the summed CVD benefits of these dietary factors are attributable only to effects on SBP and LDL-C, when in reality other pathways of benefit likely exist.

Lastly, we compared the observed vs. estimated risk using findings from the PREDIMED trial, a large RCT evaluating the effects of two overall dietary patterns on CVD incidence [53]. The estimated risk reductions were calculated by combining the observed differences in individual dietary components achieved in the trial with our estimated quantitative effects for each dietary component, assuming multiplicative effects of each individual component.

## Results

### Dietary factors with evidence for causality

We identified 10 foods and 7 nutrients with probable or convincing evidence for causal effects on specific cardiometabolic outcomes (Table 1). Among different criteria, the strength of association was most variable, and coherence, temporality, and biologic gradient were least variable (Table 2).

**Table 1:** Dietary factors and cardiometabolic outcomes with probable or convincing evidence for an etiologic relationship^1^.

Dietary Risk Factor	Cardiovascular Outcomes	Metabolic Outcomes
**Foods**		
Low fruits^2^	CHD, ischemic stroke, hemorrhagic stroke	
Low vegetables^3^	CHD, ischemic stroke, hemorrhagic stroke	
Low beans/legumes^3^	CHD	
Low nuts/seeds	CHD	Diabetes
Low whole grains	CVD, CHD	Diabetes
High red meats, unprocessed^4^		Diabetes
High processed meats^5^	CHD	Diabetes
Low fish/seafood	CHD (fatal)	
Low yogurt		Diabetes
High sugar-sweetened beverages	CHD	Diabetes, BMI^6^
**Nutrients**		
Low polyunsaturated fats (replacing either carbohydrates or saturated fats)^7^	CHD	
Low seafood omega-3 fats^8^	CHD (fatal)	
High *trans*-fats	CHD	
Low dietary fiber	CVD, CHD, stroke	Diabetes
High glycemic load	CHD, stroke	Diabetes
High dietary sodium	CVD (fatal), systolic BP^9^	
Low dietary potassium	Stroke	

^1^ See Table 2 for details on assessments of causality of each relationship.

^2^ Excluding 100% juices.

^3^ Excluding vegetable juices, starchy vegetables such as potatoes or corn, and salted or pickled vegetables. Because certain beans/legumes (e.g., black beans, lentils) were commonly included as vegetables in many of the identified studies, the etiologic effects identified for vegetables should be considered as representing the effect of vegetables including beans/legumes. We also evaluated etiologic effects of beans/legumes separately.

^4^ Beef, lamb, or pork; excluding poultry, fish, eggs, and processed meat.

^5^ Any meat preserved by smoking, curing, or salting or addition of chemical preservatives, such as bacon, salami, sausages, hot dogs, or processed deli or luncheon meats, and excluding fish or eggs.

^6^ In addition to the effect of sugar-sweetened beverages (SSBs) on adiposity (body mass index, BMI), evidence from prospective studies suggested an additional, BMI-independent effect of SSBs on incidence of type 2 diabetes and coronary heart disease (CHD).

^7^ Reported effects are nearly identical for polyunsaturated fats replacing carbohydrates or saturated fats.

^8^ Eicosapentaenoic acid (EPA) + docosahexaenoic acid (DHA).

^9^ We identified concordant evidence for direct effects on fatal cardiovascular disease (CVD) and systolic blood pressure (BP).

**Table 2:** Grading of evidence for etiologic effects of specific dietary factors on cardiometabolic outcomes^1^.

Dietary Factor	Cardiometabolic Outcome	Strength	Consistency	Temporality	Coherence	Specificity	Analogy	Plausibility	Biological Gradient	Experiment
**Foods**										
Fruits	CHD	+	++	+++	+++	++	+++^5^	+++	+++	++^23^
	Ischemic stroke	++	+++	+++	+++	++	+++^5^	+++	+++	++^23^
	Hemorrhagic stroke	+++	+++	+++	+++	++	+++^5^	+++	+++	++^23^
Vegetables	CHD	+	++	+++	+++	++	+++^5^	+++	+++	++^23^
	Ischemic stroke	++	++	+++	+++	++	+++^5^	+++	+++	++^23^
	Hemorrhagic stroke	++	++	+++	+++	++	+++^5^	+++	+++	++^23^
Beans/legumes	CHD	+++	+++	+++	+++	+++	+++^5^	+++	+++	++
Nuts/seeds	CHD (fatal)	+++	+++	+++	+++	++	+++^5^	+++	+++	+++
	CHD (non-fatal)	+++	+++	++	+++	++	+++^5^	+++	+++	+++
	Diabetes	++	+++	+++	+++	++	+++^5^	+++	+++	++
Whole grains	CVD	+	+++	+++	+++	++	+++^6^	+++	+++	+++^24^
	CHD	+	+++	+++	+++	++	+++^6^	+++	+++	+++^24^
	Diabetes	++	+++	+++	+++	++	+++^6^	+++	+++	+++^24^
Red meats, unprocessed	Diabetes	++	+++	+++	+++	+++	+++^7^	++	+++	++
Processed meats	CHD	+++	+++	+++	+++	++	+++^8^	+++	+++	+++
	Diabetes	+++	+++	+++	+++	++	+++^7^	++	+++	++
Yogurt	Diabetes	++	++	+++	+++	+++	++^9^	++	+++	++^25^
Sugar-sweetened beverages	Body mass index	+++^4^	+++	+++	+++	+++	+++^10^^,^ ^11^	+++	+++	+++
	CHD	++	+++	++	+++	++	++^10^	++	+++	++
	Diabetes	+++	+++	+++	+++	+++	+++^11^	+++	+++	+++
**Nutrients**										
PUFAs	CHD	+	++	+++	+++	+++	+^12^	+++	+++	+++
Seafood ω-3s (fish/seafood)^2^	CHD (fatal)	++	+++	+++	+++	+++	+++^13^	+++	+++^22^	+++
*Trans*-fats	CHD	+++	+++	+++	+++	+++	++^14^	+++	+++	+++
Dietary fiber	CVD	+++	+++	+++	+++	++	+++^15^	+++	+++	+++
	CHD	+++	+++	+++	+++	++	+++^15^	+++	+++	+++
	Stroke	++	+++	+++	+++	++	+++^15^	+++	+++	++
	Diabetes	++	+++	+++	+++	++	+++^16^	+++	+++	+++
Glycemic load	CHD	+++^3^	+++^3^	+++	+++	++	++^10^	++	++^21^	++
	Stroke	++	+++	+++	+++	++	+^17^	++^20^	++^21^	++
	Diabetes	+	++	+++	+++	++	+++^11^	+++	++^21^	+++
Sodium	Systolic BP	++^4^	+++	+++	+++	+++	+^19^	+++	+++	+++
	CVD (fatal)	++	++	+++	+++	++	+++^18^	+++	+	+++
Potassium	Stroke	++	+++	+++	+++	+++	+++^18^	+++	+++	+++

^1^ To score each Bradford-Hill criterion, the following general principles were utilized, focusing on evidence from meta-analyses of prospective cohort studies and/or randomized controlled trials: +++ Consistent evidence from several well-designed studies with relatively few limitations; ++ Consistent evidence from several studies but with some important limitations; + Emerging evidence from a few studies or conflicting results from several studies;—criterion not met. Definitions for each of the nine criteria and adaptations to the general scoring system were as follows: **Strength:** magnitude of association, including RRs for protective factors of >0.9 (+), 0.8–0.89 (++), or <0.8 (+++); and for harmful factors, of <1.11 (+), 1.25 (++), and >1.25 (+++). Since magnitude is directly dependent on both the selected serving size and frequency of consumption, we utilized serving sizes most similar to standard dietary guidelines and frequencies of consumption representing modest, standardized differences in intake (e.g., 1 serving/d of fruit) that are easily communicated and could be feasibly achieved by an intervention. **Consistency**: association is repeatedly observed in different populations and circumstances, including ≥80% of included study-specific estimates being in the expected direction (+++); ≥60 - <80% (++); ≥40 - <60% (+); and <40% (not meeting criteria). **Temporality:** exposure precedes outcome. Because all evidence was based on longitudinal studies, this was a necessary criterion (+++); when relatively few overall studies were available (<5), we graded this criterion conservatively as ++. **Coherence:** interpretation of association does not conflict with known natural history and biology of the disease, for example based on pathways of disease occurrence and laboratory findings on the dietary factor. **Specificity:** exposure linked to a specific outcome. Because many nutritional factors can plausibly have diverse effects and influence multiple outcomes, scoring was based on three principles: 1) dietary factor influences a mechanism/pathways known to cause the outcome; 2) dietary factor not associated with multiple other, unrelated non-communicable diseases (e.g., multiple cancers, chronic obstructive pulmonary disease (COPD)); 3) dietary association has additional specificity within the set of cardiometabolic outcomes (coronary heart disease (CHD), stroke, diabetes mellitus). **Analogy:** based on the effects of similar factors on the disease outcome; see detailed footnotes below. **Plausibility:** association supported by one or more credible biological mechanisms. **Biological gradient:** exposure and outcome are related by a monotonic dose-response curve. **Experiment:** association is also supported by evidence from randomized controlled trials on intermediate risk factors (or, less commonly, disease outcomes) plus supportive laboratory studies.

^2^ Given their common sources, these factors were evaluated together based on studies of fish/seafood, dietary long-chain omega-3 fats, and fish oil supplements.

^3^ In secondary stratified analyses, +++ for women, and—for men (main effect: null).

^4^ Effect size does not correspond to relative risk, but comparison with effect sizes on body mass index or blood pressure (BP) for other lifestyle-based interventions. For BP, the overall scored strength reflects the average of +++ for older adults, blacks, and hypertensives; and + for healthy, white, younger adults.

^5^ Based on analogies with other minimally processed, higher fiber, phytochemical rich foods.

^6^ Based on analogies with other less-processed foods, dietary fiber, and glycemic load.

^7^ Based on analogies to processed meats (or unprocessed red meats), blood ferritin levels, and hemochromatosis.

^8^ Based on analogies to sodium.

^9^ Based on analogies to probiotics in relation to weight gain.

^10^ Based on analogies to other poor-quality carbohydrates in relation to both CHD and weight gain.

^11^ Based on analogies to other poor-quality carbohydrates in relation to both diabetes mellitus and weight gain.

^12^ Based on analogies to vegetable oils in relation to CHD and cardiovascular risk factors.

^13^ Based on analogies to fish.

^14^ Based on analogies to other dietary fats.

^15^ Based on analogies to other higher-quality carbohydrates and other fiber-rich foods such as nuts, fruits, and vegetables.

^16^ Based on analogies to other higher-quality carbohydrates in relation to both diabetes mellitus and weight gain.

^17^ Based on analogies to other poor-quality carbohydrates in relation to diabetes mellitus, and diabetes as a risk factor for stroke.

^18^ Based on analogies to other lifestyle-related and nonlifestyle-related blood pressure interventions and to foods high in sodium (e.g., processed meats).

^19^ Based on analogies to potassium.

^20^ Based on insulin resistance/diabetes mellitus pathways.

^21^ Several individual studies show dose response; no published dose-response meta-analyses.

^22^ Between 0 and 250 mg/d; meta-analyses suggest no major additional benefits for fatal CHD above 250 mg/d.

^23^ Because while strong and consistent evidence from trials of dietary patterns rich in fruits and vegetables, few trials separately evaluated only fruits or vegetables.

^24^ Based on overall effects of carbohydrate quality, including studies of dietary fiber and glycemic load; much less evidence for benefits of whole grains independent of dietary fiber and glycemic load.

^25^ Based on findings for yogurt and weight gain (animal studies, human cohorts) and for probiotics and weight gain (animal and human experiments).

Our systematic searches to evaluate etiologic effects for these 17 foods and nutrients identified 896 potentially relevant meta-analyses or reviews articles, of which 23 were finally included in our estimates (Table A & Figure A in S1 File), including 1 de novo meta-analysis for 4 diet-disease relationships (Text E & Tables B-C in S1 File). We did not find sufficient probable or convincing evidence of causal etiologic effects on cardiometabolic diseases of many other dietary factors of interest, for example dietary cholesterol, plant omega-3 fats, monounsaturated fats, eggs, poultry, tea, coffee, or cocoa.

### Etiologic effects on cardiovascular diseases

Sixteen of the identified dietary factors had evidence for causal effects on CVD (Table 1). Among different clinical events, fruits, fish, and fiber were most frequently studied in relation to CHD (16 cohorts each) (Table 3). The total numbers of people in each meta-analysis of clinical events ranged from about 140,000 for *trans*-fats and CHD to about 820,000 for fruits and CHD. The largest number of total events was for processed meats and CHD (21,308 events); the fewest, for fruits or vegetables and hemorrhagic stroke (1,535 events). Across the meta-analyses for CVD, the median age at event was 61.1 years (range: 50 to 72.2 years). Relative risks were generally modest, ranging from 0.73–0.95 per daily serving of protective foods, and 1.12–1.37 for harmful foods. Some of the larger effects were for fruits and hemorrhagic stroke (RR:0.73 per daily serving), nuts/seeds and fatal CHD (0.76 per 4 servings/week), beans/legumes and CHD (0.77 per daily serving), and processed meats and CHD (1.37 per daily serving). Dietary sodium increased BP with a monotonic dose-response, with identified heterogeneity in this effect by age, race, and hypertensive status; with consistent evidence for higher risk of fatal CVD comparing high vs. low intakes. Conversely, dietary potassium was linked to lower risk of stroke, with a RR of 0.87 per 1,000 mg/d.

**Table 3:** Estimates of etiologic effects of dietary factors and risk of cardiovascular diseases and type 2 diabetes mellitus^1^.

Dietary Factor	Outcome	Studies in Each Meta-analysis^2^	Source	No. of Subjects	No. of Events	Unit of RR^3^	RR (95% CI)^3^	Statistical Heterogeneity
**Foods**								
Fruits^4^	↓ CHD	16 cohorts (22 estimates)	Gan, 2015 [71]	817,977	13,786	per 1 serving/d (100 g/d)	0.94 (0.91, 0.98)	I^2^ = 31.7% p = 0.08
	↓ Ischemic stroke	9 cohorts (10 estimates)	*De novo* meta-analysis^5^ [72–79]	329,204	5,517	per 1 serving/d (100 g/d)	0.88 (0.83, 0.93)	I^2^ = 77.1% p<0.001
	↓ Hemorrhagic stroke	5 cohorts (7 estimates)	*De novo* meta-analysis^5^ [74–77]	175,035	1,535	per 1 serving/d (100 g/d)	0.73 (0.62, 0.87)	I^2^ = 81.4% p<0.001
Vegetables ^6^	↓ CHD	9 cohorts	Gan, 2015 [71]	761,612	13,135	per 1 serving/d (100 g/d)	0.95 (0.92, 0.98)	I^2^ = 35.6% p = 0.07
	↓ Ischemic stroke	9 cohorts (10 estimates)	*De novo* meta-analysis^5^ [72–75, 77–79]	329,204	5,515	per 1 serving/d (100 g/d)	0.83 (0.75, 0.93)	I^2^ = 89.9% p<0.001
	↓ Hemorrhagic stroke	5 cohorts (7 estimates)	*De novo* meta-analysis^5^ [74–77, 79]	175,035	1,535	per 1 serving/d (100 g/d)	0.83 (0.72, 0.96)	I^2^ = 30% p = 0.20
Beans/legumes	↓ CHD	5 cohorts	Afshin, 2014 [80]	198,904	6,514	per 1 serving/d (100 g/d)	0.77 (0.65, 0.90)	I^2^ = 0.2% p = 0.41
Nuts/seeds	↓ CHD (fatal)	1 RCT and 5 cohorts	Afshin, 2014 [80]	206,114	6,749	per 4 servings/wk (4 oz/wk)	0.76 (0.69, 0.84)	I^2^ = 27.2% p = 0.23
	↓ CHD (non-fatal)	1 RCT and 3 cohorts	Afshin, 2014 [80]	141,390	2,101	per 4 servings/wk (4 oz/wk)	0.78 (0.67, 0.92)	I^2^ = 0.0% p = 0.46
	↓ Diabetes	1 RCT and 5 cohorts	Afshin, 2014 [80]	230,216	13,308	per 4 servings/wk (4 oz/wk)	0.87 (0.81, 0.94)	I^2^ = 21.6% p = 0.27
Whole grains	↓ CVD	7 cohorts (9 estimates)^7^	New GLST^8^ of Mellen 2008 [54]	285,217	7,005	per 1 serving/d (50 g/d)	0.91 (0.86, 0.97)	I^2^ = 84.0% p<0.001
	↓ CHD	6 cohorts	New GLST^8^ of Mellen 2008 [54]	281,633	4,593	per 1 serving/d (50 g/d)	0.97 (0.94, 0.99)	I^2^ = 75.5% p = 0.001
	↓ Diabetes	10 cohorts	Aune 2013 [59]	385,868	19,791	per 1 serving/d (50 g/d)	0.88 (0.83, 0.93)	I^2^ = 82% p = <0.0001
Red meats, unprocessed	↑ Diabetes	9 cohorts (10 estimates)	Pan, 2011 [81]	442,101	28,228	per 1 serving/d (100 g/d)	1.19 (1.04, 1.37)	I^2^ = 93% p<0.001
Processed meats	↑ CHD	5 cohorts (6 estimates)	Micha, 2010 [82]	614,062	21,308	per 1 serving/d (50 g/d)	1.37 (1.11, 1.68)	I^2^ = 76.2% p = 0.001
	↑ Diabetes	8 cohorts (9 estimates)	Pan, 2011 [81]	371,492	26,256	per 1 serving/d (50 g/d)	1.51 (1.25, 1.83)	I^2^ = 94.3% p<0.001
Fish/Seafood^9^	↓ CHD (fatal)	16 cohorts (17 estimates)	Zheng, 2012 [83]	315,812	4,472	per 15 g/d (~1–100 g- serving/wk)	0.94 (0.90–0.98)	I^2^ = 63.1 p<0.005
Yoghurt	↓ Diabetes	9 cohorts	Chen, 2014 [84]	408,096	32,995	per 1 serving/d (8 oz/d, 244 g/d)	0.82 (0.70, 0.96)	I^2^ = 65.3 p = 0.003
Sugar-sweetened beverages	↑ BMI (when baseline BMI <25 kg/m^2^)^10^	3 cohorts	Mozaffarian, 2011 [55]	120,877	n/a	per 1 serving/d (8 oz/d)^*111*^	0.10 kg/m^2^ (0.05, 0.15)	not reported
	↑ BMI (when baseline BMI ≥25 kg/m^2^)^10^	3 cohorts	Mozaffarian, 2011 [55]	120,877	n/a	per 1 serving/d (8 oz/d)^11^	0.23 kg/m^2^ (0.14, 0.32)	not reported
	↑ Diabetes (BMI-adjusted)^10^	17 cohorts	Imamura, 2015 [85]	464,937	38,253	per 1 serving/d (8 oz/d)^11^	1.27 (1.10, 1.46)	I^2^ = 73%
	↑ CHD (BMI-adjusted)^9^	4 cohorts	Xi, 2015 [86]	173,753	7,396	per 1 serving/d (8 oz/d)^11^	1.17 (1.10, 1.24)	I^2^ = 0.0% p = 0.79
**Nutrients**								
PUFA replacing Carbs^12^	↓ CHD	9 cohorts (12 estimates)	Farvid, 2014 [58]	262,612	12,198	per 5%E/d	0.90 (0.85, 0.94)	I^2^ = 47.3% p = 0.04
PUFA replacing SFA^12^	↓ CHD	8 cohorts (11 estimates)	Farvid, 2014 [58]	262,612	12,198	per 5%E/d	0.91 (0.87, 0.96)	I^2^ = 55.9% p = 0.01
Seafood omega-3 fats^13^	↓ CHD (fatal)	4 RCTs and 15 cohorts	Mozaffarian 2006 [87]	363,003	5,951	per 100 mg/d	0.85 (0.79, 0.92)	not reported
*Trans*-fats^14^	↑ CHD	4 cohorts	Mozaffarian, 2006 [88]	139,836	4,965	per 2% %E/d	1.23 (1.11, 1.37)	not reported
Dietary fiber^15^	↓ CVD	10 cohorts	Threapleton, 2013 [89]	1,279,690	19,869	per 20 g/d	0.76 (0.70, 0.84)	I^2^ = 45%
	↓ CHD	12 cohorts	Threapleton, 2013 [89]	1,039,572	11,282	per 20 g/d	0.76 (0.68, 0.85)	I^2^ = 33%
	↓ Stroke	7 cohorts	Threapleton, 2013 [90]	324,640	9,257	per 20 g/d	0.81 (0.70, 0.95)	I^2^ = 59%
	↓ Diabetes	5 cohorts	Yao, 2014 [91]	157,336	3,029	per 30 g/d	0.76 (0.65, 0.88)	not reported
Glycemic load^16^	↑ CHD	9 cohorts (13 estimates)	Mirrahimi, 2014 [92]	262,891	11,319	high vs. low	1.23 (1.06, 1.42)	I^2^ = 52% p = 0.02
	↑ Stroke	6 cohorts (9 estimates)	Cai, 2015 [93]	222,308	2,951	high vs. low	1.19 (1.05, 1.36)	I^2^ = 5.0% p = 0.39
	↑ Diabetes	17 cohorts (30 estimates)	Bhupathiraju, 2014 [94]	698,589	46,115	high vs. low	1.13 (1.08, 1.17)	I^2^ = 26.4% p = 0.09
Sodium^17^^,^ ^18^	↑ CVD (fatal)	11 cohorts (16 estimates)	Poggio, 2015[95]	220,249	9,628	high vs. low	1.12 (1.06, 1.19)	I^2^ = 57.6% p = 0.002
	↑ SBP, main effect, white, age 50, normotensives^18^	103 RCTs (107 estimates)	Mozaffarian, 2014 [24]	6,970	NA	per 2,300 mg/d (100 mmol/d)	3.74 mm Hg (5.18, 2.29)	not reported
	↑ SBP, additional effect per year of age < or > 50^18^						0.105 mm Hg (0.164, 0.047)	
	↑ SBP, additional effect among Blacks^18^						2.49 mm Hg (4.85, 0.13)	
	↑ SBP, additional effect among hypertensives^18^						1.87 mm Hg (3.63, 0.12)	
Potassium^17^	↓ Stroke	9 cohorts (11 estimates)	D’Elia, 2011 [96]	233,606	7,066	per 1,000 mg/d (25.7 mmol/d)	0.87 (0.79, 0.94)	I^2^ = 55% p = 0.01

^1^ Dietary factors with probable or convincing evidence, based on the Bradford-Hill criteria for assessing causality [11], for etiologic effects on cardiometabolic outcomes including coronary heart disease (CHD), stroke, cardiovascular disease (CVD), type 2 diabetes, body mass index (BMI), or systolic blood pressure (SBP).

^2^ Number of estimates can be higher than the number of studies if more than one arm in a randomized controlled trial or if estimates were separately reported by sex or age in prospective cohort studies.

^3^ Based on published or *de novo* dose-response meta-analyses of prospective cohorts or randomized trials. Meta-analyses were evaluated based on design, number of studies and events, definition of dietary exposure and disease outcomes, statistical methods, evidence of bias, and control for confounders. Relative risks (RRs) were standardized across individual studies per uniform servings of intake. When necessary, original data were extracted from individual studies to perform *de novo* dose-response meta-analyses using all available data by means of generalized least squares (GLST in STATA) for trend estimation. Effect sizes are relative risks (RRs) (95% confidence intervals (CIs)) except for sugar-sweetened beverage (SSB) effects on BMI (absolute in kg/m^2^) and sodium effects on SBP (absolute in mm Hg). Effect sizes correspond to the relationship between increased consumption of each dietary target per unit of RR and respective change in cardiometabolic risk (directionality in risk: ↑ increased, ↓ decreased). Proportional effects of major risk factors on cardiometabolic outcomes vary by age, with an inverse log-linear age association [22]. We derived age specific RRs for diet-cardiometabolic disease relationships based on the age patterns of RRs for metabolic risk factors and incident cardiometabolic disease events (see Figure B in S1 File) [22]. Except as indicated (SSBs, sodium), we did not identify sufficient evidence for effect modification by other factors beyond age, e.g. race, obesity, or overall diet quality.

^4^ Excluding 100% juices.

^5^ All four of these *de novo* meta-analyses were performed for consumption of fruits and vegetables and stroke subtypes due to absence of recent published meta-analyses; all details are provided in S1 File.

^6^ Excluding vegetable juices, starchy vegetables such as potatoes or corn, and salted or pickled vegetables. Because certain beans/legumes (e.g., black beans, lentils) were commonly included as vegetables in many of the identified studies, the etiologic effects identified for vegetables should be considered as representing the effects of vegetables including beans/legumes. We also evaluated etiologic effects of beans/legumes separately.

^7^ When a trial did not report an effect for total CVD separately (n = 3 cohorts), CHD and stroke estimates from each trial were first pooled using fixed effects (n = 2 cohorts), or the CHD estimate was used in place of CVD when that was the only reported outcome (n = 1 cohort).

^8^ Data were re-extracted from all original investigations identified in the meta-analysis to assess dose-response using two-step generalized least squares for trend estimation [18, 19].

^9^ Etiologic effects are limited to fatal CHD only due to absence of probable or convincing evidence for benefits on nonfatal CHD events. Benefits for were identified up to 3.5 servings/week of fish/seafood and 250 mg/d of eicosapentaenoic acid (EPA) + docosahexaenoic acid (DHA), with little evidence for additional benefits at higher intakes.

^10^ Available evidence suggests that SSBs increase risk through effects on both BMI and additional BMI-independent effects on type 2 diabetes and CHD [22, 23].

^11^ Depending on study-specific assumptions, use of UK or US conversion factors, and study weighting, the serving size is in this analysis could also be 8.7–9.1 oz.

^12^ Reported effects are nearly identical for polyunsaturated fats (PUFA) replacing carbohydrates (Carbs) or saturated fats (SFA).

^13^ Linear reduction in risk observed until 250 mg/day, with little evidence for additional benefits at higher intakes.

^14^ The overall causal effect was based on 4 cohorts; the final RR (95% CI) used herein was very similar but based on the isocaloric replacement of trans-fats with an equal distribution of SFA, monounsaturated fats (MUFA), and PUFA based on a meta-analysis of 2 cohorts.

^15^ Possible evidence for larger effects at intakes above 20 g/d.

^16^ Glycemic load is calculated as the glycemic index of a food multiplied by its carbohydrate content. Higher values reflect both higher glycemic index and higher quantities of refined grains, starches, and sugars. We also identified evidence for causal effects of dietary fiber. Glycemic load and dietary fiber each overlap with foods in this Table including fruits, vegetables, beans/legumes, nuts/seeds, and whole grains.

^17^ Assessed by 24h dietary recall, food frequency questionnaire, or 24h urine excretion.

^18^ Available evidence suggests that sodium increases mortality from CHD, stroke, and other BP-related cardiovascular diseases through effects on SBP [22, 24]. For every year above or below age 50, there was 0.105 mm Hg (95% CI: 0.047, 0.164) larger or smaller BP reduction, respectively. Effects on CVD vs. SBP were separately identified and are not independent (i.e., effects on CVD are at least partly mediated by SBP effects).

### Etiologic effects on diabetes

Only 8 identified dietary factors had probable or convincing evidence for causal effects on diabetes (Table 1), including protective effects of nuts/seeds, whole grains, yogurt, and dietary fiber, and harms of unprocessed red meats, processed meats, SSBs, and glycemic load. SSBs and glycemic load were most frequently studied (17 cohorts each) (Table 3). Processed meats had the strongest estimated effect, with 1.51 RR per daily serving; other foods had more modest effects, such as 0.82 and 0.88 RR per daily serving of yogurt and whole grains, respectively. SSBs had a small but statistically significant etiologic effect on body weight, with smaller effects in normal weight (per daily serving, 0.10 kg/m^2^ increase in BMI) vs. overweight or obese (0.23 kg/m^2^) individuals.

### Heterogeneity in etiologic effects

Proportional effects of most cardiovascular risk factors decline with age (inverse age association), likely related to competing risks, while absolute risk differences increase with age due to increased baseline risk [22]. We evaluated and found similar log-linear inverse associations by age for etiologic effects of major diet-related cardiometabolic risk factors [22]. We therefore applied the mean proportional differences in RRs across 6 age groups (25–34, 35–44, 45–54, 55–64, 65–74, 75+ y) for these risk factors to the dietary RRs (S6 Text & Figure B in S1 File). For both major cardiometabolic risk factors and most diet-disease relationships with sufficient evidence,[22, 54–59] we identified similar RRs by sex. One exception was glycemic load and CHD, for which stronger effects were suggested in women. For effects of SSBs on BMI, we identified and incorporated effect modification by baseline BMI, with larger effects among overweight compared with normal weight individuals [55]. For effects of sodium on SBP, we identified and incorporated joint effect modification by age, race, and hypertensive status [24].

### Evidence for optimal intakes

Based on risk as well as feasibility and consistency, we characterized optimal intakes for each dietary factor (Table 4). Potential choices of optimal ranges were broadest for dietary sodium, with differing observed optimal intakes from different CVD outcomes ranging from 614 to 2,391 mg/d [46, 60, 61] and from different major dietary guidelines ranging from 1,200 to 2,400 mg/d [33, 62–65]. Based on all available evidence, we identified a conservative optimal intake level of 2,000 mg/d as previously described [24], consistent with WHO guidelines [64].

**Table 4:** Data sources and identified optimal intake levels of specific dietary factors for reducing cardiometabolic diseases^1^.

Dietary Factor *(standardized serving size)*	Observed intake levels associated with lowest disease risk in meta-analyses (health outcome)	Observed mean national intakes in 2010^2^	Recommended intakes by major dietary guidelines^3^	Optimal mean population intake^4^
**FOODS**				
**Fruits**^5^ *(100 g/serving)*	2.4 servings/d (CHD) 3.4 servings/d (ischemic stroke) 2.1 servings/d (hemorrhagic stroke)	Top 3 countries: Barbados: 410 g/d (4.1 servings/d) Jamaica: 315 g/d (3.2 servings/d) Malaysia: 299 g/d (3.0 servings/d)	DGA 2015: 2 cups/d AHA 2020: ≥4.5 cups/d (fruits and vegetables)	3 (100 g) servings/d
**Vegetables (including beans/legumes)**^6^ *(100 g/serving)*	3.7 servings/d (CHD) 3.4 servings/d (ischemic stroke) 1.5 servings/d (hemorrhagic stroke)	Top 3 countries: Lao PDR: 364 g/d (3.6 servings/d) Bhutan: 302 g/d (3.0 servings/d) Taiwan: 293 g/d (2.9 servings/d)	DGA 2015: 1.8 cups/d (excluding starchy vegetables) AHA 2020: ≥4.5 cups/d (fruits and vegetables)	4 (100 g) servings/d
**Beans/legumes** *(100 g/serving)*	4.2 servings/wk (CHD) (0.6 servings/d)	Top 3 countries: Brazil: 194 g/d (1.9 servings/d) Colombia: 137 g/d (1.4 servings/d) Mexico: 103 g/d (1.0 serving/d)	DGA 2015: 1 ½ cups/wk (0.2 servings/d)	1 (100 g) serving/d
**Nuts/seeds** *(1 oz [28*.*35 g]/serving)*	5.0 servings/wk (fatal CHD) 5.2 servings/wk (non-fatal CHD) 4.9 servings/wk (diabetes)	Top 3 countries: Malaysia: 74 g/d (18.3 servings/wk) Lebanon: 24 g/d (6.0 servings/wk) UK: 15 g/d (3.7 servings/wk)	DGA 2015: 5 oz/wk (including soy products) AHA 2020: ≥4 servings/wk^7^	5 (1 oz) servings/wk
**Whole grains** *(50 g/serving)*	2.5 servings/d (CVD) 2.5 servings/d (CHD) 3.0 servings/d (diabetes)	Top 3 countries: Germany: 128 g/d (2.6 servings/d) Barbados: 118 g/d (2.4 servings/d) Australia: 93 g/d (1.9 servings/d)	DGA 2015: ≥3 (1 oz) servings/d (≥1.7 servings/d) AHA 2020: ≥3 (1 oz) servings/d (≥1.7 servings/d)	2.5 (50 g) servings/d
**Red meats, unprocessed** *(100 g/serving)*	0.19 servings/d (diabetes) (1.3 servings/wk)	Bottom 3 countries: Indonesia: 12 g/d (0.8 servings/wk) Armenia: 15 g/d (1.0 servings/wk) Georgia: 15 g/d (1.0 servings/wk)	DGA 2015: 26 oz/wk (lean meat, poultry and eggs) (7.4 servings/wk)	1 (100 g) serving/wk
**Processed meats** *(50 g/serving)*	0.07 serving/d (CHD) (0.5 servings/wk) 0.11 serving/d (diabetes) (0.8 servings/wk)	Bottom 3 countries: Iran: 2.5 g/d (0.4 servings/wk) Korea: 3.1 g/d (0.4 servings/wk) China: 3.3 g/d (0.5 servings/wk)	DGA 2015: Choose fresh lean meat, rather than processed meat AHA 2020: none or ≤2 servings/wk	0
**Fish/Seafood** *(100 g/serving)*	3 servings/wk (fatal CHD) 6.5 servings/wk (total stroke)	Top 3 countries: Japan: 105 g/d (7.4 servings/wk) Korea: 73 g/d (5.1 servings/wk) Iceland: 66 g/d (4.6 servings/wk)	DGA 2015: 8 oz/wk (2 servings/wk) AHA 2020: ≥2 (3.5 oz) servings/wk (preferably oily fish) (≥2 servings/wk)	3.5 (100 g) servings/wk
**Yoghurt** *(8 oz (244 g)/serving)*	2.6 servings/wk (diabetes)	Not available	DGA 2015: 3 cups/d of dairy products (21 cups/wk)	2.5 (8 oz) servings/wk
**Sugar-sweetened beverages** *(8 oz (236*.*5 g)/serving)*	0 servings/d (body mass index) 0.017 serving/d (diabetes)	Bottom 3 countries: China: 14 g/d (0.06 servings/d) Iran: 24 g/d (0.1 servings/d) Poland: 24 g/d (0.1 servings/d)	DGA 2015: <10%E from added sugars AHA 2020: ≤450 kcal (36 oz)/wk (≤0.6 servings/d)	0
**NUTRIENTS**				
**Polyunsaturated fats replacing carbohydrates or saturated fats** *(%E)*	5.1%E (CHD, cohorts) 14.9%E (CHD, randomized trials)	Top 3 countries, PUFA: Bulgaria: 11.3%E Lebanon: 9.3%E Hungary: 8.9%E Bottom 3 countries, SFA: Japan: 7.0%E Mexico: 7.4%E Barbados: 7.6%E	DGA 2015: <10%E from saturated fat replaced with unsaturated especially polyunsaturated fats FAO 2010: 6–11%E from PUFA	11%E ^9^
**Seafood omega-3 fats** *(mg/d)*	250 mg/d (fatal CHD)	Top 3 countries: Barbados: 1,191 mg/d Iceland: 1,120 mg/d Japan: 993 mg/d	DGA 2015: 250 mg/d^8^	250 mg/d
***Trans*-fats** *(%E)*	0%E (CHD)	Bottom 3 countries: Barbados: 0.2%E Finland: 0.4%E Italy: 0.5%E	DGA 2015: As low as possible WHO: <1%E [97]	0.5%E^10^
**Dietary fiber** *(g/d)*	23.8 g/d (CVD) 22.8 g/d (CHD) 19.4 g/d (stroke) 30.0 g/d (diabetes)	Top 3 countries: Barbados: 28 g/d Mexico: 26 g/d Bulgaria: 25 g/d	DGA 2015: 22.4–33.6 g/d (depending on gender & age group)	30 g/d
**Sodium** *(mg/d)*	614 mg/d (lower SBP)^11^ [60] 1,500 mg/d (reduced BP in randomized trials) [46] 1,787 mg/d (CHD mortality) [61] 2,245 mg/d (stroke mortality) [61] 2,391 mg/d (stroke)	5 nations with mean intakes at or below 2,000 mg/d: Jamaica, Colombia, South Africa, Mexico, Iran	UK NICE: <1,200 mg/d [62] AHA 2020: <1,500 mg/d [63] WHO: 2,000 mg/d [64] DGA 2015: <2,300 mg/d UK FSA: <2,400 mg/d [65]	2,000 mg/d^12^
**Potassium** *(mg/d)*	4,136 mg/d (stroke)	Not available	DGA 2015: 4,700 mg/d WHO: ≥3,500 mg/d [98]	4,500 mg/d

^1^ Building up on our work in the 2010 Nutrition and Chronic Diseases Expert Group (NutriCoDE) in which we evaluated optimal intake levels for cardiometabolic outcomes including coronary heart disease (CHD), stroke, type 2 diabetes, body mass index (BMI), and systolic blood pressure (SBP) [10]. For both studies of clinical outcomes and national surveys of dietary intakes, we used standardized servings to account for any variability in serving sizes across studies or countries. Thus, the characterized RR’s are accurate for the listed serving size. For populations with smaller or larger serving sizes, the RR’s should be appropriately adjusted.

^2^ Based on nationally representative, individual-level dietary surveys using optimal dietary metrics among both adult men and women in our 2010 NutriCoDE Global Dietary Database [27–31] and other sources [32]; adjusted to 2000 kcal/d.

^3^ For an average intake of 2,000 kcal/d.

^4^ For an average energy intake of 2,000 kcal/d. The optimal mean levels for the population were determined based on risk (observed levels at which lowest disease risk occurs), feasibility (observed national consumption levels globally), and consistency (with other assessments in major dietary guidelines) [33, 34]. The plausible population distribution of consumption (SD) around the optimal population mean was determined to be ±10% of the mean, based on the average SD for diet-related metabolic risk factors [3, 36–39]. We could not comparably identify optimal intake levels of glycemic load due to absence of global data on mean intakes in most nations and of recommended levels in major dietary guidelines.

^5^ Excluding 100% juices.

^6^ Excluding vegetable juices, starchy vegetables such as potatoes or corn, and salted or pickled vegetables.

^7^ Including beans/legumes.

^8^ Based on eicosapentaenoic acid (EPA) + docosahexaenoic acid (DHA) in common fish varieties, the DGA 2015 calculates that 1,750 mg/wk (250 mg/d) would be in concordance with the recommended fish intake of 8 oz/wk.

^*9*^ 12%E in earlier analyses [3, 25, 29]. Lowered to 11% based on the present updated review of available evidence on optimal levels and considering observed national intakes and major dietary guidelines.

^*10*^ Non-zero value to account for natural ruminant sources, for which probable or convincing evidence of causal effects on cardiometabolic outcomes was not identified.

^*11*^ Based on ecologic evidence; these values are the mean 24-hour urine sodium excretion across the 4 populations with lowest levels (Brazil, Yanomano and Xingu, Papua New Guinea, Kenya) in the Intersalt Study [60].

^*12*^ As previously described in detail, we did not incorporate a potential U-shaped relationship with risk due to the linear dose-response effect of dietary sodium on BP, the log-linear effect of BP on cardiovascular disease (CVD), the absence of plausible biologic rationale for increased risk with sodium reduction, at least to 2,000 mg/d, and the plausible source of bias that could explain the U-shaped relationships observed in some, but not all, prior observational studies [24].

AHA 2020, American Heart Association 2020 Strategic Impact Goals [35]; DGA, Dietary Guidelines for Americans [33]; FAO, United Nations Food and Agricultural Organization [34]; PUFA, Polyunsaturated fats; SFA, Saturated fats; UK FSA, Food Standards Agency; UK NICE, UK National Institute for Health and Clinical Excellence; WHO, World Health Organization.

### Assessment of validity and bias

Because our risk estimates were mostly derived from observational studies of individual dietary components, we performed several validity analyses to compare our estimated etiologic effects to other lines of evidence (Table 5, Tables D-F in S1 File). Evaluating cohort studies of dietary patterns and incident CHD, the estimated vs. observed risks were generally similar. Our estimated etiologic effects did not appreciably overestimate benefits in any study; largest differences were seen in studies of Western dietary patterns (in which our estimated etiologic effects underestimated the observed harms) and in one Greek dietary pattern study (in which our estimated etiologic effects underestimated the observed benefit). Based on changes in BP and LDL-C in dietary pattern feeding trials, the observed effects of individual dietary components were similar to our estimated etiologic effect for that dietary component, except for whole grains for which our estimated etiologic effect was smaller than that predicted by BP and LDL-C changes; and for fish for which our estimated etiologic effect on CHD death was larger than that predicted by BP and LDL-C changes alone. Finally, based on clinical events in a large randomized primary prevention trial, the observed vs. estimated relative risk reductions were similar, except for modest overestimation of benefits based on our etiologic effects in the mixed nuts group.

**Table 5:** Validity analyses comparing the observed relative risks for CHD based on evidence from prospective observational studies and randomized trials of dietary patterns versus the estimated relative risks for CHD based on the present analysis of individual dietary components.

	Observed Relative Risk for CHD ^1^	Estimated Relative Risk for CHD ^2^
**Prospective cohort studies evaluating associations of overall dietary patterns with incident CHD** ^3^		
Health Professionals Study—Prudent diet (average of all quintiles, with lowest quintile as the reference) [40]	0.82	0.78
Health Professionals Study—Western diet (average of all quintiles, with lowest quintile as the reference) [40]	1.29	1.17
Nurse’s Health Study—Prudent diet (average of all quintiles, with lowest quintile as the reference) [41]	0.82	0.80
Nurse’s Health Study—Western diet (average of all quintiles, with lowest quintile as the reference) [41]	1.20	1.10
Nurse’s Health Study—Mediterranean diet (average of all quintiles, with lowest quintile as the reference) [42]	0.84	0.81
EPIC-Greek (per 2 units diet score increase) [43]	0.78	0.90
SUN-Spain (per 2 units diet score increase) [44]	0.74	0.75
**Randomized controlled feeding trials evaluating effects of overall dietary patterns on BP and LDL-C** ^4^		
Fruits, serving/d (100 g/d)	0.93	0.94
Vegetables, serving/d (100 g/d)	0.93	0.95
Nuts and seeds, serving/wk (1 oz [28.35 g]/wk)	0.93	0.93
Whole grains, serving/d (50 g/d)	0.88	0.97
Fish, serving/d (100 g/d)	0.87	0.66
Red meat, serving/d (100 g/d)	1.17	1.17
Dietary fiber, 20 g/d	0.77	0.76
**Randomized clinical trial evaluating effects of an overall dietary pattern on incident CHD** [53, 99] ^5^		
Mediterranean diet + extra-virgin olive oil vs. placebo	0.80	0.77
Mediterranean diet + mixed nuts vs. placebo	0.74	0.62
Combined groups	0.77	0.69

^1^ Values are the observed relative risks (RRs) in these long-term prospective observational studies or randomized trials of dietary patterns.

^2^ Values are the estimated RRs based on the reported differences in intakes of individual dietary factors across each category of the diet pattern study and our estimated quantitative effects for these individual dietary factors (Table 2), assuming a multiplicative relation of proportional effects of individual components. Not all dietary factors in Table 2 were included due to insufficient reporting of differences in these components across studies of dietary patterns. We focused on foods and excluded overlapping nutrients (e.g., we included whole grains, fruits, and vegetables; and excluded fiber, glycemic load). We also assumed no benefits from differences in other dietary factors (e.g., coffee) in the dietary pattern for which we had not determined a causal etiologic effect.

^3^ Because the observed relative risks in most of these cohorts were based on serial dietary measures with time-varying updating, the predicted relative risks for each dietary factor were adjusted for comparability for regression dilution bias due to the observed changes over time of each dietary factor in these cohorts. See Table D in S1 File for more details.

^4^ For randomized controlled feeding trials of dietary patterns and cardiovascular risk factors, we performed inverse-variance-weighted meta-regression across all of the treatment arms of three large, well-established dietary pattern trials [45–47] to estimate the independent effects of five different dietary components, when consumed as part of an overall dietary pattern, on systolic blood pressure (SBP) and LDL-cholesterol (LDL-C). We evaluated achieved dietary changes in fruits, vegetables, nuts, whole grains, and fish simultaneously as independent variables in the meta-regression, with changes in SBP or LDL-C as the dependent variable. For each dietary factor, we then calculated how the identified change in SBP and LDL-C from the meta-regression would alter cardiovascular risk, based on the established relationship between SBP and LDL-C and clinical events [48–52], assuming independent, multiplicative effects of SBP and LDL-C on risk. These observed effects, calculated based only on how each dietary factor altered SBP and LDL-C in randomized controlled feeding trials of diet patterns, were then compared to our estimated etiologic effect on coronary heart disease (CHD) events for that dietary factor (Table 2). See Table E in S1 File for more details.

^5^ We compared the observed vs. predicted risk in a large randomized clinical trial evaluating the effects of two overall dietary patterns on incidence of cardiovascular events [53]. A similar analysis was previously reported using 2010 NutriCode RR’s [99]; the values here are based on the updated RR’s in the current investigation (Table 2). The predicted risk reductions were calculated by combining the observed differences in individual dietary components achieved in the trial with our estimated quantitative effects, assuming multiplicative effects of each individual component. Because we had not identified sufficient studies to quantify etiologic effects of extra-virgin olive oil, to enable comparison we imputed potential effects of extra-virgin olive oil from our estimated relative risk for polyunsaturated vegetable fats. See Table F in S1 File for more details.

## Discussion

This systematic evaluation of the evidence for effects of dietary habits on CHD, stroke, and diabetes identified and quantified probable or convincing etiologic effects and optimal consumption levels for 10 foods and 7 nutrients. Generally, minimally processed, bioactive-rich foods like fruits, vegetables, nuts/seeds, beans/legumes, and whole grains had protective effects, whereas certain more highly processed foods such as processed meats and SSBs had harmful effects. Other identified protective dietary factors were characterized by relatively unique attributes, such as fish/seafood and long-chain omega-3s (linked to lower risk of fatal CHD) or yogurt (containing active probiotics; linked to lower risk of diabetes) [12]. Fewer etiologic relationships were identified for isolated nutrients, and these were generally consistent with the findings for foods: for example, lower risk with dietary fiber and higher risk with additives such as *trans*-fats and sodium. Notably, many identified findings were specific for particular cardiometabolic outcomes: e.g., low fruit intake was identified as an etiologic risk factor for cardiovascular diseases, but not diabetes; unprocessed red meat and low yogurt intakes, as etiologic risk factors for diabetes, but not CHD or stroke; and low long-chain omega-3 intake, as an etiologic risk factor for fatal CHD, but not nonfatal CHD, stroke, or diabetes. To our knowledge, these novel findings provide the most updated, comprehensive estimates of quantitative effects of specific dietary factors on cardiometabolic disease burdens.

Whereas our evaluation for causality was based on diverse types of evidence [10], our estimates of quantitative etiologic effects mostly relied on prospective observational studies. While such studies represent a reasonable study design for evaluating long-term effects of lifestyle (as compared with pharmaceutical drugs), the results could be biased by residual confounding, particularly from other correlated dietary habits. Yet, the results of separate validity analyses, each examining estimated effects of individual dietary components as compared to observational studies or RCTs of dietary patterns, suggested low likelihood of large magnitudes of bias in our quantified etiologic effects.

Interestingly, the majority of the identified causal factors represented food groups, rather than isolated nutrients. These results are consistent with advances in nutritional science that suggest a greater relevance of foods, rather than nutrient-based metrics, for risk of chronic diseases [12, 33, 66]. Exceptions included polyunsaturated fats, representing certain vegetables, nuts/seeds, and vegetable oils; long-chain omega-3 fats, representing seafood; and dietary fiber and potassium, representing intakes of whole foods such as fruits, vegetables, nuts/seeds, and whole grains. The other identified nutrients with evidence for etiologic effects were sodium and *trans*-fats—industrial additives that can be increased or decreased in any otherwise similar food—and glycemic load, representing higher intakes of refined starches and sugars. These new findings add to a growing evidence base that emphasizes the importance of food-based diet quality in general, and minimally processed, bioactive-rich foods in particular, as key priorities for reducing burdens of cardiometabolic diseases.

Our conservative approach did not identify sufficient accumulated evidence for probable or convincing causal effects on cardiometabolic endpoints of other promising dietary factors, e.g., plant omega-3 fats, coffee, tea, cocoa. The present findings represent an assessment of the current state of evidence, and undoubtedly continuing advances in science—e.g., better dietary assessment, biomarker measures, nutrigenomics, metabolomics, personalized nutrition, other technological advances—will lead to future identification and refinement of additional important etiologic dietary factors and mechanistic pathways, for instance including polyphenols, other trace bioactives, branched chain fatty acids, and the microbiome.

Our standardized assessment of feasible optimal intake levels, informed primarily by observed levels linked to lowest risk of clinical events, provide additional new evidence to inform dietary guidelines, policy targets, and assessments of disease burdens. The identified optimal intake levels were generally similar to major dietary guidelines, supporting validity of our approach. These results do not imply assumptions about practicality or potential pace of achieving such optimal intakes for all countries, which will vary based on local cultural, economic, and political considerations. Yet, changes in national policies can induce brisk changes in dietary habits, cardiometabolic risk factors, and disease rates [67, 68], and systems approaches utilizing school, workplace, economic, built environment, and media/education strategies can effectively alter diets in populations [69, 70]. The present results on optimal intakes can be considered a set of benchmarks to quantify disease risk and inform policy priorities in different nations.

Using evidence published through 2007, Mente and colleagues reviewed evidence for etiologic effects of dietary factors on CHD; this work included only 4 Bradford-Hill criteria, compared with 9 in our investigation; and did not evaluate stroke or diabetes, evidence for optimal intake levels, or validity analysis to assess bias [8]. Others have reviewed, in narrative fashion [7], the published evidence on diet and cardiometabolic diseases, but without quantitative assessments of etiologic dose-responses, optimal consumption, or potential bias. This is the first study, to our knowledge, to systematically evaluate and quantify the current evidence for both etiologic effects and optimal levels of multiple dietary components for major cardiometabolic endpoints including CHD, stroke, and diabetes.

Our study has several strengths. We formally evaluated evidence for causality independently and in duplicate based on established Bradford-Hill criteria and assessed whether such evidence was at least probable or convincing. We quantified etiologic effects and optimal levels based on published or new meta-analyses of available evidence; including determination of dose-responses per standardized serving sizes where possible. We evaluated heterogeneity in etiologic effects by underlying individual characteristics such as age and sex. Importantly, the potential for over- or underestimation of identified effects was assessed in separate validity analyses incorporating data from long-term cohorts and randomized trials of dietary patterns.

Potential limitations should be considered. Dietary assessment in prospective cohort studies can be imperfect due to incomplete memory, questionnaire limitations, and changes in dietary habits over time; each of these factors would generally attenuate risk estimates, causing underestimation of etiologic effects. Conversely, some of the individual studies in these meta-analyses utilized serial measurements of diet, which would tend to reduce such misclassification. We did not identify sufficiently reliable data on temporal dietary changes to correct for regression dilution bias over time; our investigation identifies a need to generate such evidence across multiple cohorts and world regions. Outcome ascertainment (e.g. of CHD, diabetes) varied across studies and would be prone to error; because we focused on prospective studies, such misclassification would most often be random with respect to exposure and lead to smaller magnitudes of etiologic effects. We did not assess study quality, country, or year of publication for individual reports within each meta-analysis; we cannot exclude that differences in these factors might influence findings. Our validity analyses represented qualitative comparisons, not formal statistical tests. We limited our final estimates to dietary factors with strongest evidence, excluding many other dietary components which may influence cardiometabolic health. We did not grade strength of evidence for *absence* of health effects; a recent narrative review included some qualitative conclusions on this [12].

In sum, our novel findings provide a quantitative summary of the current evidence for causality, etiologic effects, and optimal intakes of individual dietary factors in relation to cardiometabolic disease. These findings facilitate assessment of diet-related disease burdens, investigation of comparative effectiveness and cost-effectiveness of individual and policy-level dietary interventions, and design of program priorities and prevention strategies to reduce diet-related cardiometabolic diseases.

## Supporting information

S1 File **Grading** **the Evidence for Causality**
**Text A.** Criteria for grading the evidence for etiologic effects of specific dietary factors on cardiometabolic outcomes.
**Literature** **Searches for Published Meta-analyses**
**Text B.** Searches for identifying meta-analyses of the effect of specified dietary risk factors on cardiometabolic diseases.
**Table A.** Search results, per each search strategy based on types of articles.
**Figure A.** Screening and selection process of meta-analyses evaluating etiologic effects of diet-disease relationships for dietary factors with probable or convincing evidence for effects on cardiometabolic diseases.
**De** **Novo Meta-Analyses of Fruit and Vegetable Intake and Incident Stroke**
**Text C.** Protocol for de novo meta-analyses of fruit and vegetable intake and incident stroke.
**Text D.** Search terms used to identify published prospective cohort studies examining the fruit/vegetable and stroke relationship that were published after previous fruit and vegetable meta-analyses.
**Text E.** Search results of published prospective cohort studies examining the fruit/vegetable and stroke relationship.
**Table B.** Summary results of included cohort studies in de novo meta-analysis on fruit and vegetable intake and ischemic stroke.
**Table C.** Summary results of included cohort studies in de novo meta-analysis on fruit and vegetable intake and hemorrhagic stroke.
**Etiologic** **Effects of Dietary Factors on Cardiometabolic Disease Risk**
**Text F.** Heterogeneity in etiologic effects.
**Figure B.** Age-specific relative risks for fruit intake and coronary heart disease risk.
**Validity** **Analyses**
**Table D.** Comparison of relative risks for CHD observed in prospective cohort studies of dietary patterns and estimated based on NutriCoDE relative risks for individual dietary factors.
**Table E.** Comparison of relative risks for CHD calculated based on changes in systolic blood pressure and LDL-cholesterol in randomized controlled feeding trials of dietary patterns vs. estimated relative risks based on NutriCoDE relative risks for individual dietary factors.
**Table F.** Comparison of relative risks for CHD observed in a large randomized clinical trial of dietary patterns vs. estimated relative risks based on NutriCoDE relative risks for individual dietary factor.
(DOCX)

S2 File PRISMA checklist. (DOC)

## References

[1] GBD 2013 Mortality and Causes of Death Collaborators. Global, regional, and national age-sex specific all-cause and cause-specific mortality for 240 causes of death, 1990–2013: a systematic analysis for the Global Burden of Disease Study 2013. Lancet. 2015;385(9963):117–71. Epub 2014/12/23. 10.1016/S0140-6736(14)61682-2 25530442 PMC4340604

[2] UN General Assembly. United Nations high-level meeting on noncommunicable disease prevention and control. NCD summit to shape the international agenda New York2011. http://www.who.int/nmh/events/un_ncd_summit2011/en/.

[3] Lim SS, Vos T, Flaxman AD, Danaei G, Shibuya K, Adair-Rohani H, et al A comparative risk assessment of burden of disease and injury attributable to 67 risk factors and risk factor clusters in 21 regions, 1990–2010: a systematic analysis for the Global Burden of Disease Study 2010. Lancet. 2012;380(9859):2224–60. Epub 2012/12/19. 10.1016/S0140-6736(12)61766-8 23245609 PMC4156511

[4] US Burden of Disease Collaborators. The state of US health, 1990–2010: burden of diseases, injuries, and risk factors. JAMA: the journal of the American Medical Association. 2013;310(6):591–608. Epub 2013/07/12. 10.1001/jama.2013.13805 23842577 PMC5436627

[5] Forouzanfar MH, Alexander L, Anderson HR, Bachman VF, Biryukov S, Brauer M, et al Global, regional, and national comparative risk assessment of 79 behavioural, environmental and occupational, and metabolic risks or clusters of risks in 188 countries, 1990–2013: a systematic analysis for the Global Burden of Disease Study 2013. Lancet. 2015;386(10010):2287–323. Epub 2015/09/15. 10.1016/S0140-6736(15)00128-2 26364544 PMC4685753

[6] Micha R, Penalvo JL, Cudhea F, Imamura F, Rehm CD, Mozaffarian D. Association Between Dietary Factors and Mortality From Heart Disease, Stroke, and Type 2 Diabetes in the United States. JAMA: the journal of the American Medical Association. 2017;317(9):912–24. Epub 2017/03/08. 10.1001/jama.2017.0947 28267855 PMC5852674

[7] Fardet A, Boirie Y. Associations between food and beverage groups and major diet-related chronic diseases: an exhaustive review of pooled/meta-analyses and systematic reviews. Nutrition reviews. 2014;72(12):741–62. Epub 2014/11/20. 10.1111/nure.12153 25406801

[8] Mente A, de Koning L, Shannon HS, Anand SS. A systematic review of the evidence supporting a causal link between dietary factors and coronary heart disease. Archives of internal medicine. 2009;169(7):659–69. 10.1001/archinternmed.2009.38 19364995

[9] Michas G, Micha R, Zampelas A. Dietary fats and cardiovascular disease: putting together the pieces of a complicated puzzle. Atherosclerosis. 2014;234(2):320–8. Epub 2014/04/15. 10.1016/j.atherosclerosis.2014.03.013 24727233

[10] Micha R, Kalantarian S, Wirojratana P, Byers T, Danaei G, Elmadfa I, et al Estimating the global and regional burden of suboptimal nutrition on chronic disease: methods and inputs to the analysis. European journal of clinical nutrition. 2012;66(1):119–29. Epub 2011/09/15. 10.1038/ejcn.2011.147 21915137

[11] Hill AB. The Environment and Disease: Association or Causation? Proc R Soc Med. 1965;58:295–300. 14283879 10.1177/003591576505800503 PMC1898525

[12] Mozaffarian D. Dietary and Policy Priorities for Cardiovascular Disease, Diabetes, and Obesity: A Comprehensive Review. Circulation. 2016;133(2):187–225. Epub 2016/01/10. 10.1161/CIRCULATIONAHA.115.018585 26746178 PMC4814348

[13] Word Health Organization. The World Health Report 2002: Reducing Risks, Promoting Healthy Life. World Health Organization, 2002.

[14] World Cancer Research Fund/ American Institute for Cancer Research. Food, Nutrition, Physical Activity, and the Prevention of Cancer: a Global Perspective. Washington DC: AICR: 2007.

[15] World Cancer Research Fund/ American Institute for Cancer Research. Continuous Update Project (CUP). http://www.dietandcancerreport.org/cup/report_overview/index.php.

[16] Rehm J, Mathers C, Popova S, Thavorncharoensap M, Teerawattananon Y, Patra J. Global burden of disease and injury and economic cost attributable to alcohol use and alcohol-use disorders. Lancet. 2009;373(9682):2223–33. Epub 2009/06/30. 10.1016/S0140-6736(09)60746-7 19560604

[17] Moher D, Liberati A, Tetzlaff J, Altman DG, Group P. Preferred reporting items for systematic reviews and meta-analyses: the PRISMA statement. PLoS medicine. 2009;6(7):e1000097 Epub 2009/07/22. 10.1371/journal.pmed.1000097 19621072 PMC2707599

[18] Greenland S, Longnecker MP. Methods for trend estimation from summarized dose-response data, with applications to meta-analysis. Am J Epidemiol. 1992;135(11):1301–9. Epub 1992/06/01. 1626547 10.1093/oxfordjournals.aje.a116237

[19] Orsini N, Bellocco R, Greenland S. Generalized least squares for trend estimation of summarized dose-response data. Stata J. 2006;6(1):40–57.

[20] Higgins JP, Thompson SG. Quantifying heterogeneity in a meta-analysis. Statistics in medicine. 2002;21(11):1539–58. Epub 2002/07/12. 10.1002/sim.1186 12111919

[21] Begg CB, Mazumdar M. Operating characteristics of a rank correlation test for publication bias. Biometrics. 1994;50(4):1088–101. Epub 1994/12/01. 7786990

[22] Singh GM, Danaei G, Farzadfar F, Stevens GA, Woodward M, Wormser D, et al The age-specific quantitative effects of metabolic risk factors on cardiovascular diseases and diabetes: a pooled analysis. PloS one. 2013;8(7):e65174 Epub 2013/08/13. 10.1371/journal.pone.0065174 23935815 PMC3728292

[23] Singh GM, Micha R, Khatibzadeh S, Lim S, Ezzati M, Mozaffarian D. Estimated Global, Regional, and National Disease Burdens Related to Sugar-Sweetened Beverage Consumption in 2010. Circulation. 2015;132(8):639–66. Epub 2015/07/01. 10.1161/CIRCULATIONAHA.114.010636 26124185 PMC4550496

[24] Mozaffarian D, Fahimi S, Singh GM, Micha R, Khatibzadeh S, Engell RE, et al Global sodium consumption and death from cardiovascular causes. The New England journal of medicine. 2014;371(7):624–34. Epub 2014/08/15. 10.1056/NEJMoa1304127 25119608

[25] Wang Q, Afshin A, Yakoob MY, Singh GM, Rehm CD, Khatibzadeh S, et al Impact of Nonoptimal Intakes of Saturated, Polyunsaturated, and Trans Fat on Global Burdens of Coronary Heart Disease. Journal of the American Heart Association. 2016;5(1):e002891 Epub 2016/01/23. 10.1161/JAHA.115.002891 26790695 PMC4859401

[26] Ezzati M, Lopez AD, Rodgers A, Murray CJ. Comparative Quantification of Health Risks: Global and Regional Burden of Disease Attributable to Selected Major Risk Factors (Volumes 1 and 2). Geneva: World Health Organization, 2004.

[27] Khatibzadeh S, Saheb Kashaf M, Micha R, Fahimi S, Shi P, Elmadfa I, et al A global database of food and nutrient consumption. Bulletin of the World Health Organization. 2016;94(12):931–4. Epub 2016/12/21. 10.2471/BLT.15.156323 27994286 PMC5153920

[28] Powles J, Fahimi S, Micha R, Khatibzadeh S, Shi P, Ezzati M, et al Global, regional and national sodium intakes in 1990 and 2010: a systematic analysis of 24 h urinary sodium excretion and dietary surveys worldwide. BMJ open. 2013;3(12):e003733 Epub 2013/12/25. 10.1136/bmjopen-2013-003733 24366578 PMC3884590

[29] Micha R, Khatibzadeh S, Shi P, Fahimi S, Lim S, Andrews KG, et al Global, regional, and national consumption levels of dietary fats and oils in 1990 and 2010: a systematic analysis including 266 country-specific nutrition surveys. BMJ (Clinical research ed). 2014;348:g2272. Epub 2014/04/17. 10.1136/bmj.g2272 PMC3987052 24736206

[30] Micha R, Khatibzadeh S, Shi P, Andrews KG, Engell RE, Mozaffarian D, et al Global, regional and national consumption of major food groups in 1990 and 2010: a systematic analysis including 266 country-specific nutrition surveys worldwide. BMJ open. 2015;5(9):e008705 Epub 2015/09/27. 10.1136/bmjopen-2015-008705 26408285 PMC4593162

[31] Singh GM, Micha R, Khatibzadeh S, Shi P, Lim S, Andrews KG, et al Global, Regional, and National Consumption of Sugar-Sweetened Beverages, Fruit Juices, and Milk: A Systematic Assessment of Beverage Intake in 187 Countries. PloS one. 2015;10(8):e0124845 Epub 2015/08/06. 10.1371/journal.pone.0124845 26244332 PMC4526649

[32] Eilander A, Harika RK, Zock PL. Intake and sources of dietary fatty acids in Europe: Are current population intakes of fats aligned with dietary recommendations? European journal of lipid science and technology: EJLST. 2015;117(9):1370–7. Epub 2016/02/16. 10.1002/ejlt.201400513 26877707 PMC4736684

[33] U.S. Department of Agriculture, U.S. Department of Health and Human Services. 2015–2020 Dietary Guidelines For Americans: U.S. Department of Health and Human Services and U.S. Department of Agriculture; 2015 8th: http://health.gov/dietaryguidelines/2015/guidelines/.

[34] FAO. Fats and fatty acids in human nutrition. Report of an expert consultation. Geneva: 2010 91. 21812367

[35] Lloyd-Jones DM, Hong Y, Labarthe D, Mozaffarian D, Appel LJ, Van Horn L, et al Defining and setting national goals for cardiovascular health promotion and disease reduction: the American Heart Association's strategic Impact Goal through 2020 and beyond. Circulation. 2010;121(4):586–613. 10.1161/CIRCULATIONAHA.109.192703 20089546

[36] Danaei G, Finucane MM, Lin JK, Singh GM, Paciorek CJ, Cowan MJ, et al National, regional, and global trends in systolic blood pressure since 1980: systematic analysis of health examination surveys and epidemiological studies with 786 country-years and 5.4 million participants. Lancet. 2011;377(9765):568–77. 10.1016/S0140-6736(10)62036-3 21295844

[37] Danaei G, Finucane MM, Lu Y, Singh GM, Cowan MJ, Paciorek CJ, et al National, regional, and global trends in fasting plasma glucose and diabetes prevalence since 1980: systematic analysis of health examination surveys and epidemiological studies with 370 country-years and 2.7 million participants. Lancet. 2011;378(9785):31–40. 10.1016/S0140-6736(11)60679-X 21705069

[38] Farzadfar F, Finucane MM, Danaei G, Pelizzari PM, Cowan MJ, Paciorek CJ, et al National, regional, and global trends in serum total cholesterol since 1980: systematic analysis of health examination surveys and epidemiological studies with 321 country-years and 3.0 million participants. Lancet. 2011;377(9765):578–86. 10.1016/S0140-6736(10)62038-7 21295847

[39] Finucane MM, Stevens GA, Cowan MJ, Danaei G, Lin JK, Paciorek CJ, et al National, regional, and global trends in body-mass index since 1980: systematic analysis of health examination surveys and epidemiological studies with 960 country-years and 9.1 million participants. Lancet. 2011;377(9765):557–67. 10.1016/S0140-6736(10)62037-5 21295846 PMC4472365

[40] Hu FB, Rimm EB, Stampfer MJ, Ascherio A, Spiegelman D, Willett WC. Prospective study of major dietary patterns and risk of coronary heart disease in men. The American journal of clinical nutrition. 2000;72(4):912–21. Epub 2000/09/30. 11010931 10.1093/ajcn/72.4.912

[41] Fung TT, Willett WC, Stampfer MJ, Manson JE, Hu FB. Dietary patterns and the risk of coronary heart disease in women. Archives of internal medicine. 2001;161(15):1857–62. 11493127 10.1001/archinte.161.15.1857

[42] Fung TT, Rexrode KM, Mantzoros CS, Manson JE, Willett WC, Hu FB. Mediterranean diet and incidence of and mortality from coronary heart disease and stroke in women. Circulation. 2009;119(8):1093–100. Epub 2009/02/18. 10.1161/CIRCULATIONAHA.108.816736 19221219 PMC2724471

[43] Trichopoulou A, Bamia C, Trichopoulos D. Anatomy of health effects of Mediterranean diet: Greek EPIC prospective cohort study. BMJ (Clinical research ed). 2009;338:b2337. Epub 2009/06/25. 10.1136/bmj.b2337 PMC3272659 19549997

[44] Martinez-Gonzalez MA, de la Fuente-Arrillaga C, Lopez-Del-Burgo C, Vazquez-Ruiz Z, Benito S, Ruiz-Canela M. Low consumption of fruit and vegetables and risk of chronic disease: a review of the epidemiological evidence and temporal trends among Spanish graduates. Public health nutrition. 2011;14(12A):2309–15. Epub 2011/12/15. 10.1017/S1368980011002564 22166189

[45] Appel LJ, Moore TJ, Obarzanek E, Vollmer WM, Svetkey LP, Sacks FM, et al A clinical trial of the effects of dietary patterns on blood pressure. DASH Collaborative Research Group. The New England journal of medicine. 1997;336(16):1117–24. Epub 1997/04/17. 10.1056/NEJM199704173361601 9099655

[46] Sacks FM, Svetkey LP, Vollmer WM, Appel LJ, Bray GA, Harsha D, et al Effects on blood pressure of reduced dietary sodium and the Dietary Approaches to Stop Hypertension (DASH) diet. DASH-Sodium Collaborative Research Group. The New England journal of medicine. 2001;344(1):3–10. 10.1056/NEJM200101043440101 11136953

[47] Appel LJ, Sacks FM, Carey VJ, Obarzanek E, Swain JF, Miller ER, et al Effects of protein, monounsaturated fat, and carbohydrate intake on blood pressure and serum lipids: results of the OmniHeart randomized trial. JAMA: the journal of the American Medical Association. 2005;294(19):2455–64. 10.1001/jama.294.19.2455 16287956

[48] Di Angelantonio E, Sarwar N, Perry P, Kaptoge S, Ray KK, Thompson A, et al Major lipids, apolipoproteins, and risk of vascular disease. JAMA: the journal of the American Medical Association. 2009;302(18):1993–2000. Epub 2009/11/12. 10.1001/jama.2009.1619 19903920 PMC3284229

[49] Baigent C, Keech A, Kearney PM, Blackwell L, Buck G, Pollicino C, et al Efficacy and safety of cholesterol-lowering treatment: prospective meta-analysis of data from 90,056 participants in 14 randomised trials of statins. Lancet. 2005;366(9493):1267–78. Epub 2005/10/11. 10.1016/S0140-6736(05)67394-1 16214597

[50] Law MR, Wald NJ, Rudnicka AR. Quantifying effect of statins on low density lipoprotein cholesterol, ischaemic heart disease, and stroke: systematic review and meta-analysis. BMJ (Clinical research ed). 2003;326(7404):1423. Epub 2003/06/28. 10.1136/bmj.326.7404.1423 PMC162260 12829554

[51] Lawes CM, Rodgers A, Bennett DA, Parag V, Suh I, Ueshima H, et al Blood pressure and cardiovascular disease in the Asia Pacific region. J Hypertens. 2003;21(4):707–16. Epub 2003/03/27. 10.1097/01.hjh.0000052492.18130.07 12658016

[52] Lewington S, Clarke R, Qizilbash N, Peto R, Collins R, Prospective Studies C. Age-specific relevance of usual blood pressure to vascular mortality: a meta-analysis of individual data for one million adults in 61 prospective studies. Lancet. 2002;360(9349):1903–13. 12493255 10.1016/s0140-6736(02)11911-8

[53] Estruch R, Ros E, Salas-Salvado J, Covas MI, Corella D, Aros F, et al Primary prevention of cardiovascular disease with a Mediterranean diet. The New England journal of medicine. 2013;368(14):1279–90. Epub 2013/02/26. 10.1056/NEJMoa1200303 23432189

[54] Mellen PB, Walsh TF, Herrington DM. Whole grain intake and cardiovascular disease: a meta-analysis. Nutrition, metabolism, and cardiovascular diseases: NMCD. 2008;18(4):283–90. 10.1016/j.numecd.2006.12.008 17449231

[55] Mozaffarian D, Hao T, Rimm EB, Willett WC, Hu FB. Changes in diet and lifestyle and long-term weight gain in women and men. The New England journal of medicine. 2011;364(25):2392–404. Epub 2011/06/24. 10.1056/NEJMoa1014296 21696306 PMC3151731

[56] He FJ, Nowson CA, Lucas M, MacGregor GA. Increased consumption of fruit and vegetables is related to a reduced risk of coronary heart disease: meta-analysis of cohort studies. Journal of human hypertension. 2007;21(9):717–28. Epub 2007/04/20. 10.1038/sj.jhh.1002212 17443205

[57] He K, Song Y, Daviglus ML, Liu K, Van Horn L, Dyer AR, et al Fish consumption and incidence of stroke: a meta-analysis of cohort studies. Stroke; a journal of cerebral circulation. 2004;35(7):1538–42. Epub 2004/05/25. 10.1161/01.STR.0000130856.31468.47 15155968

[58] Farvid MS, Ding M, Pan A, Sun Q, Chiuve SE, Steffen LM, et al Dietary linoleic acid and risk of coronary heart disease: a systematic review and meta-analysis of prospective cohort studies. Circulation. 2014;130(18):1568–78. Epub 2014/08/28. 10.1161/CIRCULATIONAHA.114.010236 25161045 PMC4334131

[59] Aune D, Norat T, Romundstad P, Vatten LJ. Whole grain and refined grain consumption and the risk of type 2 diabetes: a systematic review and dose-response meta-analysis of cohort studies. European journal of epidemiology. 2013;28(11):845–58. Epub 2013/10/26. 10.1007/s10654-013-9852-5 24158434

[60] INTERSALT Cooperative Research Group. Intersalt: an international study of electrolyte excretion and blood pressure. Results for 24 hour urinary sodium and potassium excretion. Intersalt Cooperative Research Group. BMJ (Clinical research ed). 1988;297(6644):319–28. Epub 1988/07/30. 10.1136/bmj.297.6644.319 PMC1834069 3416162

[61] Aburto NJ, Ziolkovska A, Hooper L, Elliott P, Cappuccio FP, Meerpohl JJ. Effect of lower sodium intake on health: systematic review and meta-analyses. BMJ (Clinical research ed). 2013;346:f1326. Epub 2013/04/06. 10.1136/bmj.f1326 PMC4816261 23558163

[62] National Institute for Health and Clinical Excellence. Prevention of cardiovascular disease at population level (NICE public health guidance 25). London: National Institute for Health and Clinical Excellence; 2010.

[63] Whelton PK, Appel LJ, Sacco RL, Anderson CA, Antman EM, Campbell N, et al Sodium, blood pressure, and cardiovascular disease: further evidence supporting the american heart association sodium reduction recommendations. Circulation. 2012;126(24):2880–9. Epub 2012/11/06. 10.1161/CIR.0b013e318279acbf 23124030

[64] World Health Organization. WHO Guideline: Sodium intake for adults and children. Geneva: WHO; 2012. 23658998

[65] Scientific Advisory Committee on Nutrition. Salt and Health. London: The Stationery Office; 2003.

[66] Mozaffarian D, Ludwig DS. Dietary guidelines in the 21st century--a time for food. JAMA: the journal of the American Medical Association. 2010;304(6):681–2. Epub 2010/08/12. 10.1001/jama.2010.1116 20699461

[67] Zatonski W, Campos H, Willett W. Rapid declines in coronary heart disease mortality in Eastern Europe are associated with increased consumption of oils rich in alpha-linolenic acid. European journal of epidemiology. 2008;23(1):3–10. Epub 2007/10/24. 10.1007/s10654-007-9195-1 17955332

[68] Capewell S, O'Flaherty M. Rapid mortality falls after risk-factor changes in populations. Lancet. 2011;378(9793):752–3. Epub 2011/03/19. 10.1016/S0140-6736(10)62302-1 21414659

[69] Mozaffarian D, Afshin A, Benowitz NL, Bittner V, Daniels SR, Franch HA, et al Population approaches to improve diet, physical activity, and smoking habits: a scientific statement from the American Heart Association. Circulation. 2012;126(12):1514–63. Epub 2012/08/22. 10.1161/CIR.0b013e318260a20b 22907934 PMC3881293

[70] Afshin A, Penalvo J, Del Gobbo L, Kashaf M, Micha R, Morrish K, et al CVD Prevention Through Policy: a Review of Mass Media, Food/Menu Labeling, Taxation/Subsidies, Built Environment, School Procurement, Worksite Wellness, and Marketing Standards to Improve Diet. Current cardiology reports. 2015;17(11):98 Epub 2015/09/16. 10.1007/s11886-015-0658-9 26370554 PMC4569662

[71] Gan Y, Tong X, Li L, Cao S, Yin X, Gao C, et al Consumption of fruit and vegetable and risk of coronary heart disease: a meta-analysis of prospective cohort studies. International journal of cardiology. 2015;183:129–37. Epub 2015/02/11. 10.1016/j.ijcard.2015.01.077 25662075

[72] Joshipura KJ, Ascherio A, Manson JE, Stampfer MJ, Rimm EB, Speizer FE, et al Fruit and vegetable intake in relation to risk of ischemic stroke. JAMA: the journal of the American Medical Association. 1999;282(13):1233–9. Epub 1999/10/12. 10517425 10.1001/jama.282.13.1233

[73] Johnsen SP, Overvad K, Stripp C, Tjonneland A, Husted SE, Sorensen HT. Intake of fruit and vegetables and the risk of ischemic stroke in a cohort of Danish men and women. The American journal of clinical nutrition. 2003;78(1):57–64. Epub 2003/06/21. 12816771 10.1093/ajcn/78.1.57

[74] Sauvaget C, Nagano J, Allen N, Kodama K. Vegetable and fruit intake and stroke mortality in the Hiroshima/Nagasaki Life Span Study. Stroke; a journal of cerebral circulation. 2003;34(10):2355–60. Epub 2003/09/23. 10.1161/01.STR.0000089293.29739.97 14500940

[75] Larsson SC, Mannisto S, Virtanen MJ, Kontto J, Albanes D, Virtamo J. Dietary fiber and fiber-rich food intake in relation to risk of stroke in male smokers. European journal of clinical nutrition. 2009;63(8):1016–24. Epub 2009/03/26. 10.1038/ejcn.2009.16 19319150 PMC3505606

[76] Oude Griep LM, Verschuren WM, Kromhout D, Ocke MC, Geleijnse JM. Raw and processed fruit and vegetable consumption and 10-year stroke incidence in a population-based cohort study in the Netherlands. European journal of clinical nutrition. 2011;65(7):791–9. Epub 2011/03/24. 10.1038/ejcn.2011.36 21427746

[77] Nagura J, Iso H, Watanabe Y, Maruyama K, Date C, Toyoshima H, et al Fruit, vegetable and bean intake and mortality from cardiovascular disease among Japanese men and women: the JACC Study. The British journal of nutrition. 2009;102(2):285–92. Epub 2009/01/14. 10.1017/S0007114508143586 19138438

[78] Steffen LM, Jacobs DR Jr., Stevens J, Shahar E, Carithers T, Folsom AR. Associations of whole-grain, refined-grain, and fruit and vegetable consumption with risks of all-cause mortality and incident coronary artery disease and ischemic stroke: the Atherosclerosis Risk in Communities (ARIC) Study. The American journal of clinical nutrition. 2003;78(3):383–90. Epub 2003/08/26. 12936919 10.1093/ajcn/78.3.383

[79] Mizrahi A, Knekt P, Montonen J, Laaksonen MA, Heliovaara M, Jarvinen R. Plant foods and the risk of cerebrovascular diseases: a potential protection of fruit consumption. The British journal of nutrition. 2009;102(7):1075–83. Epub 2009/08/04. 10.1017/S0007114509359097 19646291

[80] Afshin A, Micha R, Khatibzadeh S, Mozaffarian D. Consumption of nuts and legumes and risk of incident ischemic heart disease, stroke, and diabetes: a systematic review and meta-analysis. The American journal of clinical nutrition. 2014;100(1):278–88. Epub 2014/06/06. 10.3945/ajcn.113.076901 24898241 PMC4144102

[81] Pan A, Sun Q, Bernstein AM, Schulze MB, Manson JE, Willett WC, et al Red meat consumption and risk of type 2 diabetes: 3 cohorts of US adults and an updated meta-analysis. The American journal of clinical nutrition. 2011;94(4):1088–96. 10.3945/ajcn.111.018978 21831992 PMC3173026

[82] Micha R, Wallace SK, Mozaffarian D. Red and processed meat consumption and risk of incident coronary heart disease, stroke, and diabetes mellitus: a systematic review and meta-analysis. Circulation. 2010;121(21):2271–83. 10.1161/CIRCULATIONAHA.109.924977 20479151 PMC2885952

[83] Zheng J, Huang T, Yu Y, Hu X, Yang B, Li D. Fish consumption and CHD mortality: an updated meta-analysis of seventeen cohort studies. Public health nutrition. 2012;15(4):725–37. Epub 2011/09/15. 10.1017/S1368980011002254 21914258

[84] Chen M, Sun Q, Giovannucci E, Mozaffarian D, Manson JE, Willett WC, et al Dairy consumption and risk of type 2 diabetes: 3 cohorts of US adults and an updated meta-analysis. BMC medicine. 2014;12:215 Epub 2014/11/26. 10.1186/s12916-014-0215-1 25420418 PMC4243376

[85] Imamura F, O'Connor L, Ye Z, Mursu J, Hayashino Y, Bhupathiraju SN, et al Consumption of sugar sweetened beverages, artificially sweetened beverages, and fruit juice and incidence of type 2 diabetes: systematic review, meta-analysis, and estimation of population attributable fraction. BMJ (Clinical research ed). 2015;351:h3576. Epub 2015/07/23. 10.1136/bmj.h3576 PMC4510779 26199070

[86] Xi B, Huang Y, Reilly KH, Li S, Zheng R, Barrio-Lopez MT, et al Sugar-sweetened beverages and risk of hypertension and CVD: a dose-response meta-analysis. The British journal of nutrition. 2015;113(5):709–17. Epub 2015/03/05. 10.1017/S0007114514004383 25735740

[87] Mozaffarian D, Rimm EB. Fish intake, contaminants, and human health: evaluating the risks and the benefits. JAMA: the journal of the American Medical Association. 2006;296(15):1885–99. 10.1001/jama.296.15.1885 17047219

[88] Mozaffarian D, Katan MB, Ascherio A, Stampfer MJ, Willett WC. Trans fatty acids and cardiovascular disease. The New England journal of medicine. 2006;354(15):1601–13. 10.1056/NEJMra054035 16611951

[89] Threapleton DE, Greenwood DC, Evans CE, Cleghorn CL, Nykjaer C, Woodhead C, et al Dietary fibre intake and risk of cardiovascular disease: systematic review and meta-analysis. BMJ (Clinical research ed). 2013;347:f6879. Epub 2013/12/21. 10.1136/bmj.f6879 PMC3898422 24355537

[90] Threapleton DE, Greenwood DC, Evans CE, Cleghorn CL, Nykjaer C, Woodhead C, et al Dietary fiber intake and risk of first stroke: a systematic review and meta-analysis. Stroke; a journal of cerebral circulation. 2013;44(5):1360–8. Epub 2013/03/30. 10.1161/STROKEAHA.111.000151 23539529

[91] Yao B, Fang H, Xu W, Yan Y, Xu H, Liu Y, et al Dietary fiber intake and risk of type 2 diabetes: a dose-response analysis of prospective studies. European journal of epidemiology. 2014;29(2):79–88. Epub 2014/01/07. 10.1007/s10654-013-9876-x 24389767

[92] Mirrahimi A, Chiavaroli L, Srichaikul K, Augustin LS, Sievenpiper JL, Kendall CW, et al The role of glycemic index and glycemic load in cardiovascular disease and its risk factors: a review of the recent literature. Current atherosclerosis reports. 2014;16(1):381 Epub 2013/11/26. 10.1007/s11883-013-0381-1 24271882

[93] Cai X, Wang C, Wang S, Cao G, Jin C, Yu J, et al Carbohydrate Intake, Glycemic Index, Glycemic Load, and Stroke: A Meta-analysis of Prospective Cohort Studies. Asia-Pacific journal of public health / Asia-Pacific Academic Consortium for Public Health. 2015;27(5):486–96. Epub 2015/01/17. 10.1177/1010539514566742 25593213

[94] Bhupathiraju SN, Tobias DK, Malik VS, Pan A, Hruby A, Manson JE, et al Glycemic index, glycemic load, and risk of type 2 diabetes: results from 3 large US cohorts and an updated meta-analysis. The American journal of clinical nutrition. 2014;100(1):218–32. Epub 2014/05/03. 10.3945/ajcn.113.079533 24787496 PMC4144100

[95] Poggio R, Gutierrez L, Matta MG, Elorriaga N, Irazola V, Rubinstein A. Daily sodium consumption and CVD mortality in the general population: systematic review and meta-analysis of prospective studies. Public health nutrition. 2015;18(4):695–704. Epub 2014/05/23. 10.1017/S1368980014000949 24848764 PMC10271831

[96] D'Elia L, Barba G, Cappuccio FP, Strazzullo P. Potassium intake, stroke, and cardiovascular disease a meta-analysis of prospective studies. Journal of the American College of Cardiology. 2011;57(10):1210–9. Epub 2011/03/05. 10.1016/j.jacc.2010.09.070 21371638

[97] Nishida C, Uauy R. WHO Scientific Update on health consequences of trans fatty acids: introduction. European journal of clinical nutrition. 2009;63 Suppl 2:S1–4. Epub 2009/05/09. 10.1038/ejcn.2009.13 19424215

[98] World Health Organization. WHO Guideline: Potassium intake for adults and children. Geneva 2012. 23617019

[99] Mozaffarian D. Mediterranean diet for primary prevention of cardiovascular disease. The New England journal of medicine. 2013;369(7):673–4. Epub 2013/08/16. 10.1056/NEJMc1306659#SA3 23944310

