# Supplementary material for: Etiologic effects and optimal intakes of foods and nutrients for risk of cardiovascular diseases and diabetes: Systematic reviews and meta-analyses from the Nutrition and Chronic Diseases Expert Group (NutriCoDE)
Source: PLoS One. 2017 Apr 27;12(4):e0175149. doi: 10.1371/journal.pone.0175149 (PMC5407851; doi:10.1371/journal.pone.0175149)
Supplement: S1 File — Grading the Evidence for Causality Text A. Criteria for grading the evidence for etiologic effects of specific dietary factors on cardiometabolic outcomes. Literature Searches for Published Meta-analyses Text B. Searches for identifying meta-analyses of the effect of specified dietary risk factors on cardiometabolic diseases. Table A. Search results, per each search strategy based on types of articles. Figure A. Screening and selection process of meta-analyses evaluating etiologic effects of diet-disease relationships for dietary factors with probable or convincing evidence for effects on cardiometabolic diseases. De Novo Meta-Analyses of Fruit and Vegetable Intake and Incident Stroke Text C. Protocol for de novo meta-analyses of fruit and vegetable intake and incident stroke. Text D. Search terms used to identify published prospective cohort studies examining the fruit/vegetable and stroke relationship that were published after previous fruit and vegetable meta-analyses. Text E. Search results of published prospective cohort studies examining the fruit/vegetable and stroke relationship. Table B. Summary results of included cohort studies in de novo meta-analysis on fruit and vegetable intake and ischemic stroke. Table C. Summary results of included cohort studies in de novo meta-analysis on fruit and vegetable intake and hemorrhagic stroke. Etiologic Effects of Dietary Factors on Cardiometabolic Disease Risk Text F. Heterogeneity in etiologic effects. Figure B. Age-specific relative risks for fruit intake and coronary heart disease risk. Validity Analyses Table D. Comparison of relative risks for CHD observed in prospective cohort studies of dietary patterns and estimated based on NutriCoDE relative risks for individual dietary factors. Table E. Comparison of relative risks for CHD calculated based on changes in systolic blood pressure and LDL-cholesterol in randomized controlled feeding trials of dietary patterns vs. estimated relative risks based on NutriCoDE rel [file pone.0175149.s001.docx]

**Dietary Etiologic Effects and Optimal Intakes – S1 Supporting Information**

[**Grading the Evidence for Causality** 2](#_Toc471379883)

[**Text A.** Criteria for grading the evidence for etiologic effects of specific dietary factors on cardiometabolic outcomes. 2](#_Toc471379884)

[**Literature Searches for Published Meta-analyses** 4](#_Toc471379885)

[**Text B.** Searches for identifying meta-analyses of the effect of specified dietary risk factors on cardiometabolic diseases. 4](#_Toc471379886)

[**Table A.** Search results, per each search strategy based on types of articles. 9](#_Toc471379887)

[**Figure A.**  Screening and selection process of meta-analyses evaluating etiologic effects of diet-disease relationships for dietary factors with probable or convincing evidence for effects on cardiometabolic diseases. 10](#_Toc471379888)

[**De Novo Meta-Analyses of Fruit and Vegetable Intake and Incident Stroke** 11](#_Toc471379889)

[**Text C.** Protocol for de novo meta-analyses of fruit and vegetable intake and incident stroke. 11](#_Toc471379890)

[**Text D.** Search terms used to identify published prospective cohort studies examining the fruit/vegetable and stroke relationship that were published after previous fruit and vegetable meta-analyses. 12](#_Toc471379891)

[**Text E.** Search results of published prospective cohort studies examining the fruit/vegetable and stroke relationship. 13](#_Toc471379892)

[**Table B.** Summary results of included cohort studies in de novo meta-analysis on fruit and vegetable intake and ischemic stroke. 14](#_Toc471379893)

[**Table C.** Summary results of included cohort studies in de novo meta-analysis on fruit and vegetable intake and hemorrhagic stroke. 16](#_Toc471379894)

[**Etiologic Effects of Dietary Factors on Cardiometabolic Disease Risk** 17](#_Toc471379895)

[**Text F.** Heterogeneity in etiologic effects. 17](#_Toc471379896)

[**Figure B.** Age-specific relative risks for fruit intake and coronary heart disease risk. 17](#_Toc471379897)

[**Validity Analyses** 18](#_Toc471379898)

[**Table D.**  Comparison of relative risks for CHD observed in prospective cohort studies of dietary patterns and estimated based on NutriCoDE relative risks for individual dietary factors. 18](#_Toc471379899)

[**Table E.** Comparison of relative risks for CHD calculated based on changes in systolic blood pressure and LDL-cholesterol in randomized controlled feeding trials of dietary patterns vs. estimated relative risks based on NutriCoDE relative risks for individual dietary factors. 20](#_Toc471379900)

[**Table F.** Comparison of relative risks for CHD observed in a large randomized clinical trial of dietary patterns vs. estimated relative risks based on NutriCoDE relative risks for individual dietary factor. 22](#_Toc471379901)

[**References** 23](#_Toc471379902)

# Grading the Evidence for Causality

## Text A. Criteria for grading the evidence for etiologic effects of specific dietary factors on cardiometabolic outcomes.

The following principles, focusing on meta-analyses of prospective cohort studies and/or randomized controlled trials, guided the scoring for each of 9 Bradford-Hill criteria: +++ Consistent evidence from several well-designed studies with relatively few limitations; ++ Consistent evidence from several studies but with some important limitations; + Emerging evidence from a few studies or conflicting results from several studies; - criterion not met. Definitions for each of the 9 criteria and adaptations to the general scoring system were as follows:

1. **Strength:** magnitude of association, including RRs for protective factors of >0.9 (+), 0.8-0.89 (++), or <0.8 (+++); and for harmful factors, of <1.11 (+), 1.25 (++), and >1.25 (+++). Since magnitude is directly dependent on both the selected serving size and frequency of consumption, we utilized serving sizes most similar to standard dietary guidelines and frequencies of consumption representing modest, standardized differences in intake (e.g., 1 serving/d of fruit) that are easily communicated and could be feasibly achieved by an intervention.
2. **Consistency**: association is repeatedly observed in different populations and circumstances, including ≥80% of included study-specific estimates being in the expected direction (+++); ≥60 - <80% (++); ≥40 - <60% (+); and <40% (not meeting criteria). (Though some other grading frameworks use statistical heterogeneity, this is not optimal to assess consistency as characterized by Bradford-Hill. Statistical measures of heterogeneity are influenced by both magnitudes of differences and also the numbers of studies and precision of each estimate. Thus, diet-disease relationships with few studies could have lack of consistency but fail to achieve statistical heterogeneity due to low power; while diet-disease relationships having many studies with high precision could exhibit statistical heterogeneity yet still be consistent in terms of their overall inference for the effect of the dietary factor on disease.)
3. **Temporality:** exposure precedes outcome. Because all evidence was based on longitudinal studies, this was a necessary criterion (+++); when relatively few overall studies were available (<5), we graded this criterion conservatively as ++.
4. **Coherence:** interpretation of association does not conflict with known natural history and biology of the disease, for example based on pathways of disease occurrence and laboratory findings on the dietary factor.
5. **Specificity:** exposure linked to a specific outcome. Because many nutritional factors can plausibly have diverse effects and influence multiple outcomes, scoring was based on three principles: 1) dietary factor influences a mechanism/pathways known to cause the outcome; 2) dietary factor not associated with multiple other, unrelated non-communicable diseases (e.g., multiple cancers, chronic obstructive pulmonary disease (COPD)); 3) dietary association has additional specificity within the set of cardiometabolic outcomes (coronary heart disease (CHD), stroke, diabetes mellitus).
6. **Analogy:** based on the effects of similar factors on the disease outcome; see detailed footnotes in Table 2.
7. **Plausibility:** association supported by one or more credible biological mechanisms.
8. **Biological gradient:** exposure and outcome are related by a monotonic dose-response curve.
9. **Experiment:** association is also supported by evidence from randomized controlled trials on intermediate risk factors (or, less commonly, disease outcomes) plus supportive laboratory studies.

Following grading of Bradford-Hill criteria, the characterization of overall sufficient probable or convincing evidence for each diet-disease relationship was based on independent review by two investigators (RM, DM) of the overall findings across the Bradford-Hill criteria,[[1](#_ENREF_1)] with additional guidance from other definitions for probable or convincing evidence of causality from the WHO and WCRF/AICR;[[2-4](#_ENREF_2)] any differences were resolved by consensus.

# Literature Searches for Published Meta-analyses

## Text B. Searches for identifying meta-analyses of the effect of specified dietary risk factors on cardiometabolic diseases.

For each identified diet-disease relationship, we performed multiple systematic searches of PubMed to identify meta-analyses of randomized controlled trials or prospective cohort studies evaluating these specific dietary factors and total cardiovascular disease, coronary heart disease including subtypes (fatal, nonfatal), stroke including subtypes (ischemic, hemorrhagic), or diabetes. For sodium and sugar-sweetened beverages, we also reviewed effects on blood pressure and obesity, respectively, based on randomized trials demonstrating primary effects on these risk pathways. We did not search for individual papers/studies across multiple dietary risk factors and outcomes, rather we only included published, peer-reviewed meta-analyses; or performed de novo meta-analyses with all methods presented. Based on our and other recent reviews,[[5](#_ENREF_5)] we did not include multiple other factors for which the initial appraisal identified one or more key limitations that would limit meeting the criteria for causality. For example, coffee was a leading candidate for additional inclusion; others included extra-virgin olive oil, monounsaturated fat, cocoa, and tea. Using the example of coffee, our recent reviews[[5](#_ENREF_5)] identified no established mechanistic evidence from multiple prior randomized controlled trials that would provide biologic plausibility to explain the relatively large reduction. Several small controlled trials have evaluated the potential effects of habitual coffee consumption on cardiometabolic risk factors, with mixed and inconsistent findings to date.[[6-8](#_ENREF_6)] A Mendelian randomization study, evaluating genetic variants linked to coffee intake, also did not find associations with any cardiovascular or metabolic risk factors.[[9](#_ENREF_9)] For extra-virgin olive oil, while ecologic studies and short-term trials of surrogate outcomes suggested benefits, relatively few long-term cohorts evaluated extra-virgin olive oil per se; and one multi-component clinical trial included extra-virgin olive oil but also other interventions precluding isolation of benefits of extra-virgin olive oil per se. While monounsaturated fat derived from plant sources is more consistently associated with lower cardiometabolic risk, monounsaturated fat derived from animal sources is not.[[5](#_ENREF_5)] Because the molecule itself (oleic acid) is identical regardless of the source, and because at least some trials in nonhuman primates suggest potential atherogenecity of monounsaturated fat (e.g., due to generation of cholesteryl oleate), we conservatively concluded that total monounsaturated fat did not meet sufficient criteria for at least probable evidence of causal benefits. We noted that this conclusion was also consistent with the report of the 2015 US Dietary Guidelines Advisory committee.[[10](#_ENREF_10)] Due to the multiple dietary factors evaluated, a formal listing of the specific reasons for the exclusion of each was not recorded.

**PubMed Search Terms**

**Limits:**

**Age:** Any

**Setting**: Any country

**Year Range**: Any

**Language**: English

**Species**: Human

**Type of Article**: Meta-Analysis [ptyp] OR Meta-Analysis [tiab] OR "Systematic Review"[tiab]

Note: if a search term exists as Mesh, then use both ([mesh] OR [tiab]); if only free-text searching (i.e., no Mesh) then use [tiab]

**Date**: May/1/2005 through May/1/2015

**Dietary Factors:**

**Foods**

Fruits

Fruit juices

Vegetables

Beans/legumes

Nuts/seeds

Whole grains

Dairy

Milk

Yogurt

Cheese

Meats

Total meats

Red meats

Processed meats

Fish

Eggs

Sugar-sweetened beverages

Coffee/ Tea

Cocoa

**Nutrients**

Dietary fatty acids

Polyunsaturated fats

Saturated fats

Seafood omega-3 fats

Plant omega-3 fats

Trans fats

Dietary cholesterol

Dietary fiber

Glycemic load

Dietary sodium

Dietary potassium

Dietary calcium

Energy

**EXPOSURE**

**Fruits**

(“fruit” [MeSH] OR “fruit” [tiab] OR “fruits” [tiab])

**Fruit Juices**

(“fruit juice” [tiab] OR “fruit juices” [tiab] OR “juice”[tiab])

**Vegetables**

(“Vegetables” [MeSH] OR “Vegetables” [tiab] OR “Vegetable” [tiab])

**Beans/legumes**

(“Bean” [tiab] OR “Beans” [tiab] OR “legumes” [tiab] OR “legumes” [tiab])

**Nuts/seeds**

(“Nuts” [MeSH] OR “Nuts” [tiab] OR “Nut” [tiab] OR “Seeds” [MeSH] OR “Seeds” [tiab] OR “Seed” [tiab])

**Whole grains**

(“Whole grains” [tiab] OR “Whole grain” [tiab])

**Dairy**

(“Dairy Products” [MeSH] OR “dairy” [tiab] OR “cheese” [MeSH] OR “cheese” [tiab] OR “yogurt” [MeSH] OR “yogurt” [tiab] OR “yoghurt” [tiab] OR “milk” [MeSH] OR “milk”[tiab])

**Unprocessed red meat and processed meat**

(“meat” [Mesh] OR “meat” [tiab])

**Fish**

(“fishes” [MeSH] OR “fishes” [tiab] OR “fish” [tiab])

**Eggs**

(“eggs” [MeSH] OR “eggs” [tiab])

**Sugar sweetened beverages**

(“Sugar-sweetened beverages” [tiab] OR “Sugar-sweetened beverage” [tiab] OR “Sugar sweetened beverages” [tiab] OR “Sugar sweetened beverage” [tiab] OR “beverages” [MeSH] OR “beverages” [tiab] OR “beverage” [tiab])

**Coffee and tea**

(“coffee” [MeSH] OR “coffee” [tiab] OR “tea” [MeSH] OR “tea” [tiab])

**Cocoa**

(“Cacao”[MeSH] OR “Cacao”[tiab] OR “cocoa” [tiab] OR (“dark”[tiab] AND “chocolate”[tiab]))

**Dietary fatty acids**

("Fatty Acids, Omega-6"[MeSH] OR "Fatty Acids, Omega-3"[MeSH] OR "Fatty Acids, Unsaturated"[MeSH] OR "Fatty Acids, Monounsaturated"[ MeSH] OR "Trans Fatty Acids"[MeSH] OR "monounsaturated"[tiab] OR "mono-unsaturated"[tiab] OR "MUFA"[tiab] OR "unsaturated"[tiab] OR "polyunsaturated"[tiab] OR "PUFA"[tiab] OR "saturated"[tiab] OR "SFA"[tiab] OR "trans-unsaturated"[tiab] OR "trans-fatty"[tiab] OR "trans fatty"[tiab] OR "trans unsaturated"[tiab] OR "trans fat"[tiab] OR "TFA"[tiab] OR "omega-6"[tiab] OR "omega-3"[tiab] OR "n-6"[tiab] OR "n-3"[tiab] OR “alpha-linolenic”[tiab] OR "oleic"[tiab] OR "linoleic"[tiab])

**Dietary cholesterol**

“Cholesterol, Dietary” [MeSH] OR “Dietary cholesterol” [tiab])

**Dietary fiber**

(“Dietary Fiber” [MeSH] OR “Dietary Fiber” [tiab] OR “Dietary Fibers” [tiab])

**Glycemic load**

(“Glycemic Load” [MeSH] OR “glycemic load” [tiab] OR “glycaemic load” [tiab] OR “glycemic index”[MeSH] OR “glycemic index”[tiab] OR “glycaemic index” [tiab])

**Dietary sodium**

(“Sodium, Dietary” [MeSH] OR “Dietary Sodium” [tiab])

**Dietary potassium**

(“Potassium, Dietary” [MeSH] OR “Dietary Potassium” [tiab])

**Dietary calcium**

((“Calcium” [MeSH] AND "diet"[MeSH)) OR (“dietary” [tiab] AND “Calcium” [tiab]))

**Energy**

(“Energy Intake” [MeSH] OR “Energy Intake” [tiab] OR “Caloric Restriction” [MeSH] OR “Caloric Restriction” [tiab])

**OUTCOME**

(“Cardiovascular Diseases” [MeSH] OR “Cardiovascular Disease” [tiab] OR “Cardiovascular Diseases” [tiab] OR “Heart Diseases” [MeSH] OR “Heart Diseases” [tiab] OR "myocardial infarction"[tiab] OR "myocardial infarctions"[tiab] OR "heart attack"[tiab] OR "heart attacks"[tiab] OR "sudden death"[tiab] OR "sudden deaths"[tiab] OR stroke[tiab] OR strokes[tiab] OR "cerebrovascular accident"[tiab] OR "cerebrovascular accidents"[tiab] OR “Diabetes Mellitus” [MeSH] OR “Diabetes Mellitus” [tiab] OR “Diabetes” [tiab] OR “Diabetes Mellitus, Type 2” [MeSH])

**PUBLICATION**

1

AND (“Meta-Analysis” [ptyp]) AND ("2005/05/01"[PDat] : "2015/05/01"[PDat]) AND Humans[Mesh] AND English[lang]))

2

AND (“Meta-Analysis” [ptyp] OR "Meta-Analysis" [tiab]) AND ("2005/05/01"[PDat] : "2015/05/01"[PDat]) AND Humans[Mesh] AND English[lang]))

3

AND (“Meta-Analysis” [ptyp] OR "Review" [ptyp]) AND ("2005/05/01"[PDat] : "2015/05/01"[PDat]) AND Humans[Mesh] AND English[lang]))

4

AND (“Meta-Analysis” [ptyp] OR “Systematic Review” [tiab]) AND ("2005/05/01"[PDat] : "2015/05/01"[PDat]) AND Humans[Mesh] AND English[lang]))

5

AND (“Meta-Analysis” [ptyp] OR “Meta-Analysis” [tiab] OR "Systematic Review" [tiab]) AND ("2005/05/01"[PDat] : "2015/05/01"[PDat]) AND Humans[Mesh] AND English[lang]))

Table A. Search results, per each search strategy based on types of articles. *^1^*

| **Risk Factor** | **Meta-Analysis[ptyp]** | **Meta-Analysis[ptyp] OR Meta-Analysis[tiab]** | **Meta-Analysis[ptyp] OR Review[ptyp]** | **Meta-Analysis[ptyp] OR "Systematic Review"[tiab]** | **Meta-Analysis[ptyp] OR Meta-Analysis[tiab] OR "Systematic Review"[tiab]** |
| --- | --- | --- | --- | --- | --- |
| **FOODS** |  | | | | |
| Fruits | 64 | 81 | 720 | 93 | 107 |
| Fruit juices | 2 | 5 | 60 | 7 | 9 |
| Vegetables | 57 | 74 | 608 | 81 | 94 |
| Beans/legumes | 12 | 17 | 90 | 13 | 17 |
| Nuts/seeds | 42 | 50 | 335 | 57 | 64 |
| Whole grains | 10 | 13 | 132 | 20 | 22 |
| Dairy | 33 | 45 | 364 | 45 | 56 |
| Meats | 30 | 44 | 198 | 39 | 50 |
| Fish | 55 | 77 | 590 | 73 | 92 |
| Eggs | 4 | 5 | 42 | 5 | 6 |
| Sugar-sweetened beverages | 71 | 91 | 571 | 91 | 108 |
| Coffee and tea | 50 | 61 | 318 | 59 | 68 |
| Cocoa | 10 | 12 | 107 | 11 | 5 |
| **NUTRIENTS** |  |  |  |  |  |
| Dietary fatty acids | 265 | 341 | 2334 | 385 | 448 |
| Dietary cholesterol | 3 | 4 | 64 | 4 | 5 |
| Dietary fiber | 29 | 37 | 275 | 41 | 46 |
| Glycemic load | 42 | 51 | 254 | 50 | 58 |
| Dietary sodium | 26 | 36 | 400 | 36 | 45 |
| Dietary potassium | 7 | 7 | 45 | 7 | 7 |
| Dietary calcium | 9 | 19 | 189 | 16 | 23 |
| Energy | 39 | 55 | 623 | 52 | 66 |
| **Total** | **860** | **1125** | **8319** | **1185** | **1396** |
| **Total (excluding duplicates)** | **575** | **727** | **5397** | **782** | **896** |

*^1^* We focused on the last strategy, i.e. including “Meta-Analysis[ptyp] OR Meta-Analysis[tiab] OR “Systematic Review"[tiab]” in our study, as the most efficient and comprehensive (see Figure A).

[ptyp]: Publication type; [tiab]: In title or abstract.

Figure A. Screening and selection process of meta-analyses evaluating etiologic effects of diet-disease relationships for dietary factors with probable or convincing evidence for effects on cardiometabolic diseases. CVD, cardiovascular disease; GLST, generalized least squares for trend estimation.

Screening

Inclusion

New meta-analysis

Identification

Meta-analyses meeting inclusion criteria

- Reported dose response for 22 diet-disease relationships (n=17)
- Did not report dose response for 5 diet-disease relationships (n=5)

De novo meta-analyses on fruits and vegetables and CVD outcomes; 4 diet-disease relationships (n=1)

De novo GLST using all categorical data from original studies for 2 diet-disease relationships (n= 1)

Articles excluded

(n = 874)

- Not relevant outcome or exposure
- Study design
- Large heterogeneity
- Selection bias

31 diet-disease relationships from 23 meta-analyses

Records identified through systematic PubMed search

(n= 896)

# De Novo Meta-Analyses of Fruit and Vegetable Intake and Incident Stroke

## Text C. Protocol for de novo meta-analyses of fruit and vegetable intake and incident stroke.

**Objective**

To systematically review and quantify the relationship between fruit and vegetable consumption and risk of incident stroke (ischemic, hemorrhagic) in adults.

**Hypothesis**

We hypothesize that dietary intake of fruits and vegetables will be associated with decreased risk of specified clinical endpoints (ischemic stroke, hemorrhagic stroke).

**Methods**

The recommendations of Preferred Reporting Items for Systematic reviews and Meta-Analyses (PRISMA) and Meta-analysis of Observational Studies in Epidemiology (MOOSE) guidelines will be followed during all stages of the design, implementation, and reporting of this meta-analysis.

**Inclusion Criteria**

1. **Study Design:** Prospective cohort studies (including nested case-control design).
2. **Follow-up Duration:** >3 months.
3. **Population:** General adult human population 18 years or older.
4. **Exposure:** Intake of fruits or vegetables, either continuous or in >2 categories of intake to allow for adequate categorization of fruit and vegetable intake.
5. **Outcome:** Incident ischemic and hemorrhagic stroke.
6. **Effect Estimate:** Studies providing multi-variate adjusted effect estimate (OR, RR, HR) and variance and information on, or sufficient information to calculate effect estimate variance. Studies also included if such information can be obtained from the author.
7. **Setting:** No restrictions on type of setting will be applied.
8. **Language:** English.
9. **Publication type:** Full-text, published, peer reviewed.
10. **Year:** Earliest available up to Jan 30^th^ 2012.

**Exclusion Criteria**

1. **Study Design:** Retrospective case-control studies.
2. **Population:** Younger than 18 years old, pregnant women, children, and specified diseased (e.g. end-stage or rare diseases) or special (e.g., vegetarians vs. non-vegetarians) populations.
3. **Exposure:** mixed healthy diet was reported, where the effect of fruit and vegetables could not be separated.
4. **Outcome:** Studies of prevalence of outcome, studies of intermediate risk factors to the outcomes of interest (e.g. lipids, hypertension).
5. **Effect Estimate:** unadjusted (crude) risk estimates only.
6. **Duplicate Publications:** When duplicate publications from the study are identified, the report on the largest number of cases for each endpoint of interest will be included.

Text D. Search terms used to identify published prospective cohort studies examining the fruit/vegetable and stroke relationship that were published after previous fruit and vegetable meta-analyses.[[11](#_ENREF_11), [12](#_ENREF_12)]

**LIMITS:**

**Age:** Any

**Setting**: Any country

**Year Range**: Any

**Language**: English

**Species**: Human

**Search query for PubMed**: (fruits OR vegetables) AND (cardiovascular OR stroke OR hemorrhage OR ischemic OR cerebral) AND (prospective OR cohort OR longitudinal OR participants)

**Date**: 01/30/2012

**Abstracts identified**: 730

## Text E. Search results of published prospective cohort studies examining the fruit/vegetable and stroke relationship.

- **730 hits**
- 547 hits from PubMed search
- 183 related citations (from 29 initially included articles)
- **22 studies included**
- Optimal: 10 (6 articles already included in Dauchet or He meta-analyses, 1 article an update of a cohort included previously)
- Fruit and vegetable intake combined: 2
- Stroke not reported separately from CVD: 10 (17 initially included, but 7 excluded upon closer examination during extraction)
- **Notable exclusions**
- Yamada et al., 2011[[13](#_ENREF_13)] included just citrus fruits. Questionnaire appears to have asked about other fruits, but total fruits not reported in this publication. PubMed search reveals no other publication on this cohort that reported total fruits.
- **Summary of studies included in following analysis (optimal and F&V combined):**
- Stroke
- 5 new cohorts not included in Dauchet or He meta-analyses
- 1 updated cohort
- CVD
- 10 cohorts (13 estimates)

## Table B. Summary results of included cohort studies in de novo meta-analysis on fruit and vegetable intake and ischemic stroke.

| **Cohort** | **Country** | **Exposure** | **Exposure Level** | **Outcome *^1^*** | **Sample Size** | **Events** | **Fruits RR (95% CI)** | **Vegetables RR (95% CI)** |
| --- | --- | --- | --- | --- | --- | --- | --- | --- |
| Health Professionals' Follow-up Study, 1999[[14](#_ENREF_14)] | USA | fruits and vegetables separately | per 100 g/d *^2^* | ischemic stroke | 38,683 | 204 | 0.92 (0.85-1.00) | 0.96 (0.88-1.05) |
| Nurses' Health Study, 1999[[14](#_ENREF_14)] | USA | fruits and vegetables separately | per 100 g/d *^2^* | ischemic stroke | 75,596 | 366 | 0.89 (0.84-0.94) | 0.94 (0.89-0.99) |
| Danish Cancer and Health Study, 2003[[15](#_ENREF_15)] | Denmark | fruits and vegetables separately | per 100 g/d | ischemic stroke | 54,506 | 266 | 0.87 (0.81-0.93) | 1.01 (0.86-1.19) |
| Nagasaki Life Span Study (men), 2003[[16](#_ENREF_16)] | Japan | fruits and green/yellow vegetables separately | per 100 g/d *^2^* | cerebral infarction | 14,966 | 348 | 0.61 (0.50-0.73) | 0.66 (0.52-0.83) |
| Nagasaki Life Span Study (women), 2003[[16](#_ENREF_16)] | Japan | fruits and green/yellow vegetables separately | per 100 g/d *^2^* | cerebral infarction | 23,471 | 572 | 0.75 (0.63-0.90) | 0.68 (0.57-0.82) |
| Alpha-Tocopherol, Beta-Carotene Cancer Prevention Study, 2009[[17](#_ENREF_17)] *^3^* | Finland | fruits and vegetables separately | per 100 g/d | cerebral infarction | 26,556 | 2,702 | 0.90 (0.86-0.95) | 0.81 (0.76-0.87) |
| Zutphen study, 1996[[18](#_ENREF_18)] | Netherlands | solid fruits and vegetables separately | per 100 g/d | stroke | 552 | 42 | 0.59 (0.39-0.90) | 0.82 (0.43-1.54) |
| Framingham study, 1995[[19](#_ENREF_19)] | USA | fruits and vegetables separately | per 100 g/d *^2^* | stroke and TIA | 832 | 97 | 0.93 (0.82-1.06) | 0.90 (0.81-1.01) |
| MORGEN study, 2011[[20](#_ENREF_20)] | Netherlands | raw fruits and raw vegetables separately *^4^* | per 100 g/d | ischemic stroke | 20,069 | 139 | 0.99 (0.81-1.22) | 0.13 (0.07-0.23) |
| Japan Collaborative Cohort Study, 2009[[21](#_ENREF_21)] | Japan | fruits and vegetables separately | per 100 g/d *^2^* | ischemic stroke | 59,485 | 362 | 0.66 (0.47-0.91) | 1.12 (0.29-4.41) |
| Atherosclerosis Risk in Communities Study, 2003[[22](#_ENREF_22)] | USA | fruits and vegetables combined *^5^* | per 100 g/d *^2^* | ischemic stroke | 11,940 | 214 | 0.96 (0.89-1.04) | 0.96 (0.89-1.04) |
| NHANES Epidemiological Follow-up Study, 2002[[23](#_ENREF_23)] | USA | fruits and vegetables combined *^5^* | per 100 g/d *^2^* | stroke | 9,608 | 888 | 0.89 (0.84-0.94) | 0.89 (0.84-0.94) |
| Finnish Mobile Clinic Health Examination Survey, 2009[[24](#_ENREF_24)] | Finland | fruits and vegetables separately | per 100 g/d *^6^* | ischemic stroke | 3,932 | F: 344  V: 342 | 0.96 (0.92-0.99) | 1.02 (0.95-1.09) |
| Pooled, random F: I^2^ = 73.0%, p < 0.001 V: I^2^ = 86.7%, p < 0.001 |  |  | per 100 g/d | ischemic and total stroke *^7^* | 340,196 | F: 6,544  V: 6,542 | 0.88 (0.84-0.93) | 0.86 (0.79-0.93) |
| Pooled, random F: I^2^ = 77.1%, p < 0.001 V: I^2^ = 89.9%, p < 0.001 |  |  | per 100 g/d | ischemic stroke *^8^* | 329,204 | F: 5,517  V: 5,515 | 0.88 (0.83-0.93) | 0.83 (0.75-0.93) |
| Pooled, random: without MORGEN *^9^* V: I^2^ = 81.4%, p < 0.001 |  |  | per 100 g/d | ischemic stroke *^8^* | 309,135 | V: 5,376 |  | 0.89 (0.82-0.97) |
| Pooled, He, random[[12](#_ENREF_12)]  (standardized to 100 g) |  |  | per 100 g/d | total stroke | 237,718 | 3,718 | 0.88 (0.85-0.90) | 0.94 (0.91-0.96) |
| Pooled, Dauchet, fixed[[11](#_ENREF_11)]  (standardized to 100 g) |  |  | per 100 g/d | ischemic stroke | F: 186,298 V: 171,332 | F: 1,184 V: 836 | 0.89 (0.86-0.92) | 0.99 (0.93-1.04) |

*^1^* If study reported estimate for ischemic stroke, then that estimate was used in analysis. If study reported estimate only for total stroke, then total stroke estimate was used in analysis.

*^2^* Reported exposure in servings/d. Assumed 100 g per serving in this analysis.

*^3^* All participants in cohort are male smokers.

*^4^* Summary estimates reported for raw and processed separately. Raw fruits selected because processed fruits included primarily citrus juice (49%) and apple juice (22%). Raw vegetables selected because processed vegetables included primarily cabbages (24%) and french beans (14%).

*^5^* Effect size reported for fruit and vegetable intake combined. Same effect size and intake used for both fruit and vegetable pooled analyses.

*^6^* Intake reported separately for men and women, but relative risk reported for men and women combined. In this analysis, calculated weighted average of the two sexes for intake.

*^7^* Analysis includes all studies.

*^8^* Analysis excludes estimates for the outcome of total stroke, i.e. Zutphen study, Framingham study, and NHANES Epidemiological Follow-up Study.

*^9^* Because the MORGEN study’s estimate for vegetables was identified as an outlier (RR: 0.13, 95% CI: 0.07, 0.23), this study was excluded in a sensitivity analysis.

## Table C. Summary results of included cohort studies in de novo meta-analysis on fruit and vegetable intake and hemorrhagic stroke.

| **Cohort** | **Country** | **Exposure** | **Exposure Level** | **Outcome** | **Sample Size** | **Events** | **Fruits RR (95% CI)** | **Vegetables RR (95% CI)** |
| --- | --- | --- | --- | --- | --- | --- | --- | --- |
| Nagasaki Life Span Study[[16](#_ENREF_16)] | Japan | fruits and green/yellow vegetables separately | per 100 g/d *^1^* | cerebral hemorrhage | 14,966 | 166 | 0.60 (0.45-0.81) | 0.82 (0.53-1.25) |
| Nagasaki Life Span Study (women)[[16](#_ENREF_16)] | Japan | fruits and green/yellow vegetables separately | per 100 g/d *^1^* | cerebral hemorrhage | 23,471 | 287 | 0.61 (0.49-0.76) | 0.84 (0.62-1.14) |
| Alpha-Tocopherol, Beta-Carotene Cancer Prevention Study[[17](#_ENREF_17)] *^2^* | Finland | fruits and vegetables separately | per 100 g/d | subarachnoid hemorrhage *^3^* | 26,556 | 196 | 0.95 (0.78-1.15) | 0.68 (0.56-0.83) |
| Alpha-Tocopherol, Beta-Carotene Cancer Prevention Study[[17](#_ENREF_17)] *^2^* | Finland | fruits and vegetables separately | per 100 g/d | intracerebral hemorrhage *^3^* | 26,556 | 383 | 0.89 (0.77-1.04) | 0.80 (0.67-0.96) |
| MORGEN study[[20](#_ENREF_20)] | Netherlands | raw fruits and raw vegetables separately *^4^* | per 100 g/d | hemorrhagic stroke | 20,069 | 45 | 0.69 (0.56-0.85) | 0.89 (0.14-5.45) |
| Japan Collaborative Cohort Study[[21](#_ENREF_21)] | Japan | fruits and vegetables separately | per 100 g/d *^1^* | hemorrhagic stroke | 59,485 | 393 | 0.49 (0.38-0.64) | 1.12 (0.61-2.05) |
| Finnish Mobile Clinic Health Examination Survey, 2009[[24](#_ENREF_24)] | Finland | fruits and vegetables separately | per 100 g/d *^5^* | intracerebral hemorrhage | 3,932 | F: 65  V: 64 | 0.93 (0.88-0.98) | 1.09 (0.83-1.43) |
| Pooled, random F: I^2^ = 86.8%, p < 0.001 V: I^2^ = 30.2%, p = 0.20 |  |  | per 100 g/d | hemorrhagic stroke *^6^* | 175,035 | F: 1,535  V:1,534 | 0.73 (0.62-0.87) | 0.83 (0.72-0.96) |
| Pooled, random F: I^2^ = 88.8%, p < 0.001 V: I^2^ = 0.0%, p = 0.53 |  |  | per 100 g/d | w/o subarachnoid *^7^* | 148,479 | F: 1,339  V:1,338 | 0.70 (0.57-0.86) | 0.88 (0.77-1.00) |

*^1^* Reported exposure in servings/d. Assumed 100 g per serving in this analysis.

*^2^* All participants in cohort are male smokers.

*^3^* Endpoint was first stroke after randomization, so no individual is included as an event for both subarachnoid hemorrhage and intracerebral hemorrhage; outcomes are mutually exclusive.

*^4^* Summary estimates reported for raw and processed separately. Raw fruits selected because processed fruits included primarily citrus juice (49%) and apple juice (22%). Raw vegetables selected because processed vegetables included primarily cabbages (24%) and french beans (14%).

*^5^* Intake reported separately for men and women, but relative risk reported for men and women combined. In this analysis, calculated weighted average of the two sexes for intake.

*^6^* Analysis includes all studies.

*^7^* Analysis excludes estimates for the outcome of subarachnoid hemorrhage, i.e. Alpha-Tocopherol, Beta-Carotene Cancer Prevention Study.

# Etiologic Effects of Dietary Factors on Cardiometabolic Disease Risk

## Text F. Heterogeneity in etiologic effects.

We quantified multivariable-adjusted effect estimates (relative risks) from recent or de novo systematic reviews and meta-analyses of prospective studies and/or clinical trials of clinical endpoints. Most meta-analyses did not stratify by sex, and those that did found no significant differences in proportional effects of dietary factors between men and women; in addition, the proportional effects of most metabolic risk factors on chronic diseases appear similar by sex.[[25-33](#_ENREF_25)] Thus, we incorporated similar proportional effects of dietary factors by sex. Conversely, our own and others’ work has demonstrated that proportional effects of major risk factors on cardiometabolic diseases vary by age, with an inverse log-linear age association.[[34-36](#_ENREF_34)] We therefore derived and utilized age-group specific RRs for diet-cardiometabolic disease relationships based on the age patterns of RRs for metabolic risk factors and incident cardiometabolic disease events.[[34-36](#_ENREF_34)] To quantify and incorporate this effect modification by age, we evaluated the proportional differences in RRs for major diet-related cardiometabolic risk factors, including systolic blood pressure (BP), body mass index (BMI), fasting plasma glucose and total cholesterol, across 6 age groups from 25-34 to 75+ years. Because proportional differences between adjacent age groups were quite similar across these four risk factors, we applied the mean proportional differences in RR by age across all risk factors to the dietary RRs, anchored at the mean age at event of each diet-disease pair. In applying these to diet, we used Monte Carlo simulations to estimate the uncertainty in the age-distributed log RRs, sampling from the distribution of log RRs at the age at event. Based on 1000 simulations, we utilized the 2.5^th^ and 97.5^th^ percentiles to derive the 95% uncertainty interval. An example is presented for fruit consumption and coronary heart disease (Figure B). With certain exceptions (e.g., sugar-sweetened beverages, sodium), we did not identify sufficient evidence for effect modification by other factors beyond age, such as race, obesity, or overall diet quality.

## Figure B. Age-specific relative risks for fruit intake and coronary heart disease risk.

**
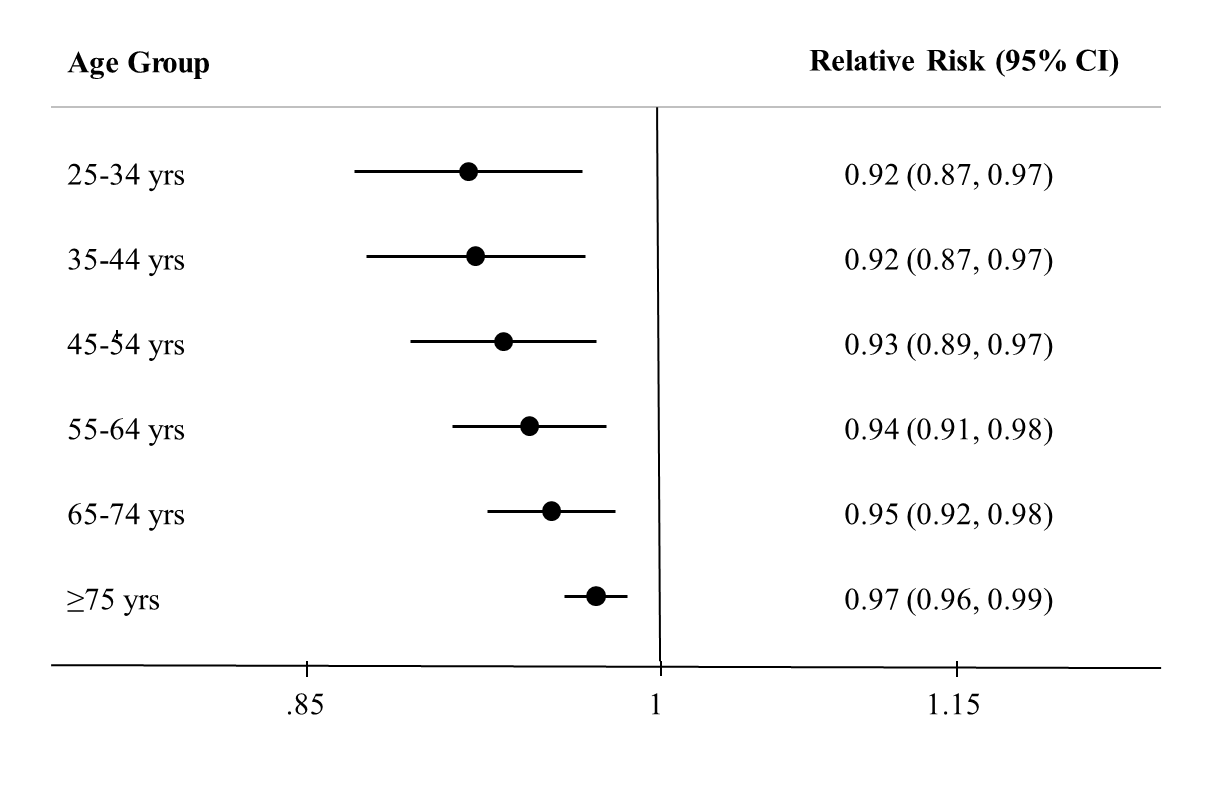
**

# Validity Analyses

Table D. Comparison of relative risks for CHD observed in prospective cohort studies of dietary patterns and estimated based on NutriCoDE relative risks for individual dietary factors. *^1^*

| **Study** | **Estimate type** | **Q2** | **Q3** | | **Q4** | | **Q5** | **Average of all quintiles** *^2^* | **Mean absolute risk difference, calculated vs. observed** *^3^* |
| --- | --- | --- | --- | --- | --- | --- | --- | --- | --- |
| Health Professionals Study – Prudent Dietary Pattern[[37](#_ENREF_37)] | Calculated RR in each quintile *^4^* | 0.86 | 0.82 | | 0.74 | | 0.63 | 0.75 | 0.07 |
|  | Calculated RR, adjusted for time *^5^* | 0.88 | 0.83 | | 0.76 | | 0.66 | 0.78 | 0.04 |
|  | Observed RR | 0.90 | 0.83 | | 0.79 | | 0.75 | 0.82 |  |
|  |  |  |  | |  | |  |  |  |
| Health Professionals Study – Western Dietary Pattern[[37](#_ENREF_37)] | Calculated RR in each quintile *^4^* | 1.12 | 1.16 | | 1.19 | | 1.30 | 1.19 | -0.10 |
|  | Calculated RR, adjusted for time *^5^* | 1.10 | 1.14 | | 1.17 | | 1.26 | 1.17 | -0.12 |
|  | Observed RR | 1.21 | 1.27 | | 1.27 | | 1.43 | 1.29 |  |
|  |  |  |  | |  | |  |  |  |
| Nurses’ Health Study – Prudent Dietary Pattern[[38](#_ENREF_38)] | Calculated RR in each quintile *^4^* | 0.91 | 0.80 | | 0.75 | | 0.66 | 0.77 | 0.05 |
|  | Calculated RR, adjusted for time *^5^* | 0.92 | 0.82 | | 0.77 | | 0.69 | 0.80 | 0.02 |
|  | Observed RR | 0.95 | 0.83 | | 0.76 | | 0.76 | 0.82 |  |
|  |  |  |  | |  | |  |  |  |
| Nurses’ Health Study – Western Dietary Pattern[[38](#_ENREF_38)] | Calculated RR in each quintile *^4^* | 1.04 | 1.07 | | 1.15 | | 1.23 | 1.12 | -0.08 |
|  | Calculated RR, adjusted for time *^5^* | 1.03 | 1.06 | | 1.13 | | 1.20 | 1.10 | -0.10 |
|  | Observed RR | 1.01 | 1.10 | | 1.26 | | 1.46 | 1.20 |  |
|  |  |  |  | |  | |  |  |  |
| Nurses’ Health Study – Mediterranean Dietary Pattern[[39](#_ENREF_39)] | Calculated RR in each quintile *^6^* | 0.93 | 0.82 | | 0.76 | | 0.72 | 0.80 | 0.04 |
|  | Calculated RR, adjusted for time *^5^* | 0.93 | 0.83 | | 0.77 | | 0.73 | 0.81 | 0.03 |
|  | Observed RR | 0.92 | 0.87 | | 0.87 | | 0.71 | 0.84 |  |
|  |  | Men | | Women | | Overall | |  |  |
| EPIC Greece – Mediterranean Dietary Pattern[[40](#_ENREF_40)] | Calculated RR per 2 unit increase *^7^* | 0.90 | | 0.91 | | 0.90 | |  | -0.12 |
|  | Observed RR | 0.81 | | 0.75 | | 0.78 | |  |  |
|  |  |  | | |  | | |  |  |
| SUN Cohort Spain – Mediterranean Dietary Pattern[[41](#_ENREF_41)] | Calculated RR per 2 unit increase *^8^* | 0.75 | | | | | |  | -0.01 |
|  | Observed RR | 0.74 | | | | | |  |  |
|  |  | | | | | | |  |  |

*^1^* The observed multivariable-adjusted relative risk (RR) in each category or per each unit of the dietary pattern was compared to the predicted effect calculated by combining the reported differences in individual dietary factors (including fruit, vegetables, whole grains, fish, processed meat, *trans* fat, polyunsaturated fat) across each category or per unit of the diet pattern with their Nutrition and Chronic Diseases Expert Group (NutriCoDE)-estimated individual quantitative effects, assuming a multiplicative relation between RRs for individual components. We primarily utilized the RRs for foods and excluded overlapping components (e.g., whole grains and fiber; or meats and saturated fats) in these analyses. The calculated RRs also assumed no benefits from changes in other dietary factors (e.g., coffee) for which we had not determined a causal etiologic effect, which could cause the observed RRs to be greater than the calculated RRs; and also incorporated the NutriCoDE threshold of optimal intake, beyond which no further benefit was assumed.

*^2^* Based on the mean of beta-coefficients (ln RR’s) across quintiles within each study.

*^3^* Based on the mean absolute risk difference of calculated vs. observed RR’s [(1-calculated RR) - (1-observed RR)]. Compared to observed RRs as the reference, positive values represent overestimation of calculated RRs, while negative values represent underestimation of calculated RRs.

*^4^* Dietary factors reported and included were fruits, vegetables, whole grains, fish, processed meat, *trans* fat, and polyunsaturated fat.

*^5^* Accounting for observed declining dietary differences over time in the dietary pattern studies in these specific cohorts.

*^6^* Dietary factors reported and included were fruit, vegetables, whole grains, omega-3s, processed and red meat, and *trans* fat.

*^7^* Dietary factors reported and included were vegetables plus legumes, fruits and nuts, fish, processed meat, and polyunsaturated fat. The dietary comparisons used were for the 75th vs. 25th percentiles.

*^8^* Dietary factors reported and included were vegetables plus legumes, fruits, fish, whole grains, nuts, processed meats, and polyunsaturated fat.

## Table E. Comparison of relative risks for CHD calculated based on changes in systolic blood pressure and LDL-cholesterol in randomized controlled feeding trials of dietary patterns vs. estimated relative risks based on NutriCoDE relative risks for individual dietary factors.

| **Dietary risk factor** | **Change in SBP (mmHg) in dietary feeding RCTs** *^1^* | **Change in LDL-C (mg/dL) in dietary feeding RCTs** *^1^* | **Predicted RR of CHD in dietary feeding RCTs, based on SBP effect** *^2^* | **Predicted RR of CHD in dietary feeding RCTs, based on LDL-C effect** *^2^* | **Multiplicative RR of CHD per serving in dietary feeding RCTs, based on joint SBP and LDL-C effects** | **Estimated RR of CHD from cohort studies**  **(NutriCoDE relative risks, see Table 2)** |
| --- | --- | --- | --- | --- | --- | --- |
| Fruits, per serving/d  (100 g/d) | -0.33 | -1.5 | 0.99 | 0.94 | 0.93 | 0.94  (0.91, 0.98) |
| Vegetables, per serving/d  (100 g/d) | -0.18 | -1.6 | 0.99 | 0.94 | 0.93 | 0.95  (0.92, 0.98) |
| Nuts/seeds, per serving/wk  (28.35 g) | -0.92 | -1 | 0.97 | 0.96 | 0.93 | 0.93  (0.91, 0.96) |
| Whole grains, per serving/d  (50 g/d) | -0.11 | -3.2 | 1.00 | 0.88 | 0.88 | 0.97  (0.94, 0.99) |
| Fish, per serving/d  (100 g/d) | N/A*^3^* | -3.4 | N/A *^3^* | 0.87 | 0.87 | 0.66  (0.50, 0.87) |
| Red meat, per serving/d  (100 g/d) | 3.20 | 1.1 | 1.12 | 1.04 | 1.17 | 1.17  (1.05, 1.30) *^4^* |
| Dietary fiber, per 20 g/d  (20 g/d) *^5^* | -3.00 | -3.9 | 0.89 | 0.86 | 0.77 | 0.76  (0.68, 0.85) |

*^1^* For systolic blood pressure (SBP), studies include OmniHeart (protein diet vs. baseline diet, carbohydrate diet vs. baseline diet, and unsaturated fat diet vs. baseline diet),[[42](#_ENREF_42)] DASH-sodium (high sodium DASH diet vs. high sodium control diet),[[43](#_ENREF_43)] and DASH (combination diet vs. control diet, fruit and vegetable diet vs. control diet).[[44](#_ENREF_44)] For LDL-C, studies include OmniHeart (protein diet vs. baseline diet, carbohydrate diet vs. baseline diet, and unsaturated fat diet vs. baseline diet), DASH-sodium (high sodium DASH diet vs. high sodium control diet, intermediate sodium DASH diet vs. intermediate sodium control diet, low sodium DASH diet vs. low sodium control diet). Results reflect pooled meta-regression models simultaneously accounting for all dietary changes in these dietary patterns trials, i.e. changes in each dietary factor in this Table.

*^2^* Based on the observed association between SBP and incident coronary heart disease (CHD) events and LDL-C and incident CHD events in large pooling projects of prospective cohort studies.[[37](#_ENREF_37), [38](#_ENREF_38), [45](#_ENREF_45)]

*^3^* Analysis of the impact of including fish in the blood pressure meta-regression model indicated that small changes in fish intake caused improbably large changes in blood pressure. Therefore, fish was not included in the blood pressure meta-regression.

*^4^* Based on prospective cohort studies, we identified evidence for an etiologic relative risk (RR) for CHD for processed meat, but not unprocessed red meat. Because these feeding studies evaluated only total meat consumption, the corresponding RR for cohort studies represents the estimated RR for total meat consumption, based on approximately 25% of total meat consumption being processed meat, a 100 g serving size, and assuming no significant etiologic effect of unprocessed red meat.

*^5^* Due to their substantial overlap, dietary fiber was excluded from meta-regression models estimating changes in SBP and LDL-C in which fruits, vegetables, nuts and seeds, and whole grains were independent variables. Likewise, fruits, vegetables, nuts and seeds, and whole grains were excluded in models in which dietary fiber was an independent variable.

NutriCoDE, Nutrition and Chronic Diseases Expert Group.

Table F. Comparison of relative risks for CHD observed in a large randomized clinical trial of dietary patterns vs. estimated relative risks based on NutriCoDE relative risks for individual dietary factor. *^1^*

| **Dietary Factor** | **Achieved change in the EVOO group, g/d** *^2^* | **Achieved change in the nut group, g/d** | **NutriCoDE -estimated effect on MI[**[**41**](#_ENREF_41)**]** | **NutriCoDE serving size, g/d** | **Calculated effect on MI in the EVOO group[**[**41**](#_ENREF_41)**]** | **Calculated effect on MI in the nut group[**[**41**](#_ENREF_41)**]** | **Calculated effect on MI in combined groups[**[**41**](#_ENREF_41)**]** |
| --- | --- | --- | --- | --- | --- | --- | --- |
| Fruits | 6.25 | 12.5 | 0.94 | 100 | 0.996 | 0.992 | 0.994 |
| Vegetables | 1.75 | 10.0 | 0.95 | 100 | 0.999 | 0.995 | 0.997 |
| Beans/legumes | 2.40 | 2.4 | 0.77 | 100 | 0.994 | 0.994 | 0.994 |
| Nuts/seeds | 3.25 | 21.0 | 0.77 *^3^* | 16.2 | 0.949 | 0.713 | 0.822 |
| Seafood n-3 fatty acids | 0.11 | 0.12 | 0.92 *^4^* | 0.1 | 0.912 | 0.905 | 0.909 |
| Extra-virgin olive oil (%E) | 4.97 | 1.08 | 0.90 *^5^* | 5.0 | 0.901 | 0.977 | 0.938 |
|  | ***Calculated Overall Effect (all six dietary factors)*** | | | | ***0.771*** | ***0.618*** | ***0.691*** |
|  | ***Observed Effect in PREDIMED*** | | | | ***0.800*** | ***0.740*** | ***0.770*** |

*^1^* For consistency with the other validity analyses (Tables S4-S5), we focused on results for CHD in the PREDIMED (Prevencion con Dieta Mediterranea) trial.[[46](#_ENREF_46)] A similar analysis was previously reported using 2010 NutriCoDE RR’s;[[47](#_ENREF_47)] the findings here are based on the updated RR’s in the current investigation (Table 2).

*^2^* Values are g/d except for extra virgin olive oil (EVOO), which is percent energy (%E).

*^3^* Assuming half of myocardial infarctions (MIs) were fatal, and half nonfatal.

*^4^* Assuming half of MI’s were fatal, as the risk reduction for marine n-3 fatty acids is specific for fatal MI, not nonfatal MI.

*^5^* Assuming effects on MI are similar to those of vegetable oil polyunsaturated fatty acids.

EVOO, extra-virgin olive oil. NutriCoDE, Nutrition and Chronic Diseases Expert Group.

# References

1. Hill AB. The Environment and Disease: Association or Causation? Proc R Soc Med. 1965;58:295-300. PMID: 14283879.

2. WHO. The World Health Report 2002: Reducing Risks, Promoting Healthy Life. World Health Organization, 2002.

3. World Cancer Research Fund/ American Institute for Cancer Research. Food, Nutrition, Physical Activity, and the Prevention of Cancer: a Global Perspective. Washington DC: AICR: 2007.

4. World Cancer Research Fund/ American Institute for Cancer Research. Continuous Update Project (CUP). Available from: <http://www.dietandcancerreport.org/cup/report_overview/index.php>.

5. Mozaffarian D. Dietary and Policy Priorities for Cardiovascular Disease, Diabetes, and Obesity: A Comprehensive Review. Circulation. 2016;133(2):187-225. Epub 2016/01/10. doi: 10.1161/CIRCULATIONAHA.115.018585. PMID: 26746178; PMCID: PMCPMC4814348.

6. Wedick NM, Brennan AM, Sun Q, Hu FB, Mantzoros CS, van Dam RM. Effects of caffeinated and decaffeinated coffee on biological risk factors for type 2 diabetes: a randomized controlled trial. Nutr J. 2011;10:93. Epub 2011/09/15. doi: 10.1186/1475-2891-10-93. PMID: 21914162; PMCID: PMCPMC3180352.

7. Ohnaka K, Ikeda M, Maki T, Okada T, Shimazoe T, Adachi M, et al. Effects of 16-week consumption of caffeinated and decaffeinated instant coffee on glucose metabolism in a randomized controlled trial. Journal of nutrition and metabolism. 2012;2012:207426. Epub 2012/11/30. doi: 10.1155/2012/207426. PMID: 23193459; PMCID: PMCPMC3502017.

8. Steffen M, Kuhle C, Hensrud D, Erwin PJ, Murad MH. The effect of coffee consumption on blood pressure and the development of hypertension: a systematic review and meta-analysis. J Hypertens. 2012;30(12):2245-54. Epub 2012/10/04. doi: 10.1097/HJH.0b013e3283588d73. PMID: 23032138.

9. Nordestgaard AT, Thomsen M, Nordestgaard BG. Coffee intake and risk of obesity, metabolic syndrome and type 2 diabetes: a Mendelian randomization study. Int J Epidemiol. 2015;44(2):551-65. Epub 2015/05/24. doi: 10.1093/ije/dyv083. PMID: 26002927.

10. Dietary Guidelines Advisory Committee. 2015 Report of the Dietary Guidelines Advisory Committee on the Dietary Guidelines for Americans: US Department of Agriculture, Agriculutural Research Service; 2015 [cited 2015]. Available from: <https://health.gov/dietaryguidelines/2015-scientific-report/>.

11. Dauchet L, Amouyel P, Dallongeville J. Fruit and vegetable consumption and risk of stroke: a meta-analysis of cohort studies. Neurology. 2005;65(8):1193-7. Epub 2005/10/26. doi: 65/8/1193 [pii]

10.1212/01.wnl.0000180600.09719.53 [doi]. PMID: 16247045.

12. He FJ, Nowson CA, MacGregor GA. Fruit and vegetable consumption and stroke: meta-analysis of cohort studies. Lancet. 2006;367(9507):320-6. Epub 2006/01/31. doi: 10.1016/S0140-6736(06)68069-0. PMID: 16443039.

13. Yamada T, Hayasaka S, Shibata Y, Ojima T, Saegusa T, Gotoh T, et al. Frequency of citrus fruit intake is associated with the incidence of cardiovascular disease: the Jichi Medical School cohort study. J Epidemiol. 2011;21(3):169-75. Epub 2011/03/11. doi: JST.JSTAGE/jea/JE20100084 [pii]. PMID: 21389640.

14. Joshipura KJ, Ascherio A, Manson JE, Stampfer MJ, Rimm EB, Speizer FE, et al. Fruit and vegetable intake in relation to risk of ischemic stroke. JAMA : the journal of the American Medical Association. 1999;282(13):1233-9. Epub 1999/10/12. PMID: 10517425.

15. Johnsen SP, Overvad K, Stripp C, Tjonneland A, Husted SE, Sorensen HT. Intake of fruit and vegetables and the risk of ischemic stroke in a cohort of Danish men and women. The American journal of clinical nutrition. 2003;78(1):57-64. Epub 2003/06/21. PMID: 12816771.

16. Sauvaget C, Nagano J, Allen N, Kodama K. Vegetable and fruit intake and stroke mortality in the Hiroshima/Nagasaki Life Span Study. Stroke; a journal of cerebral circulation. 2003;34(10):2355-60. Epub 2003/09/23. doi: 10.1161/01.str.0000089293.29739.97. PMID: 14500940.

17. Larsson SC, Mannisto S, Virtanen MJ, Kontto J, Albanes D, Virtamo J. Dietary fiber and fiber-rich food intake in relation to risk of stroke in male smokers. European journal of clinical nutrition. 2009;63(8):1016-24. Epub 2009/03/26. doi: 10.1038/ejcn.2009.16. PMID: 19319150; PMCID: PMCPMC3505606.

18. Keli SO, Hertog MG, Feskens EJ, Kromhout D. Dietary flavonoids, antioxidant vitamins, and incidence of stroke: the Zutphen study. Archives of internal medicine. 1996;156(6):637-42. Epub 1996/03/25. PMID: 8629875.

19. Gillman MW, Cupples LA, Gagnon D, Posner BM, Ellison RC, Castelli WP, et al. Protective effect of fruits and vegetables on development of stroke in men. JAMA : the journal of the American Medical Association. 1995;273(14):1113-7. Epub 1995/04/12. PMID: 7707599.

20. Oude Griep LM, Verschuren WM, Kromhout D, Ocke MC, Geleijnse JM. Raw and processed fruit and vegetable consumption and 10-year stroke incidence in a population-based cohort study in the Netherlands. European journal of clinical nutrition. 2011;65(7):791-9. Epub 2011/03/24. doi: 10.1038/ejcn.2011.36. PMID: 21427746.

21. Nagura J, Iso H, Watanabe Y, Maruyama K, Date C, Toyoshima H, et al. Fruit, vegetable and bean intake and mortality from cardiovascular disease among Japanese men and women: the JACC Study. The British journal of nutrition. 2009;102(2):285-92. Epub 2009/01/14. doi: 10.1017/s0007114508143586. PMID: 19138438.

22. Steffen LM, Jacobs DR, Jr., Stevens J, Shahar E, Carithers T, Folsom AR. Associations of whole-grain, refined-grain, and fruit and vegetable consumption with risks of all-cause mortality and incident coronary artery disease and ischemic stroke: the Atherosclerosis Risk in Communities (ARIC) Study. The American journal of clinical nutrition. 2003;78(3):383-90. Epub 2003/08/26. PMID: 12936919.

23. Bazzano LA, He J, Ogden LG, Loria CM, Vupputuri S, Myers L, et al. Fruit and vegetable intake and risk of cardiovascular disease in US adults: the first National Health and Nutrition Examination Survey Epidemiologic Follow-up Study. The American journal of clinical nutrition. 2002;76(1):93-9. Epub 2002/06/26. PMID: 12081821.

24. Mizrahi A, Knekt P, Montonen J, Laaksonen MA, Heliovaara M, Jarvinen R. Plant foods and the risk of cerebrovascular diseases: a potential protection of fruit consumption. The British journal of nutrition. 2009;102(7):1075-83. Epub 2009/08/04. doi: 10.1017/s0007114509359097. PMID: 19646291.

25. Jakobsen MU, O'Reilly EJ, Heitmann BL, Pereira MA, Balter K, Fraser GE, et al. Major types of dietary fat and risk of coronary heart disease: a pooled analysis of 11 cohort studies. The American journal of clinical nutrition. 2009;89(5):1425-32. PMID: 19211817.

26. Lawes CM, Rodgers A, Bennett DA, Parag V, Suh I, Ueshima H, et al. Blood pressure and cardiovascular disease in the Asia Pacific region. J Hypertens. 2003;21(4):707-16. Epub 2003/03/27. doi: 10.1097/01.hjh.0000052492.18130.07. PMID: 12658016.

27. Zhang X, Patel A, Horibe H, Wu Z, Barzi F, Rodgers A, et al. Cholesterol, coronary heart disease, and stroke in the Asia Pacific region. Int J Epidemiol. 2003;32(4):563-72. Epub 2003/08/13. PMID: 12913030.

28. Lawes CM, Parag V, Bennett DA, Suh I, Lam TH, Whitlock G, et al. Blood glucose and risk of cardiovascular disease in the Asia Pacific region. Diabetes care. 2004;27(12):2836-42. PMID: 15562194.

29. Ni Mhurchu C, Rodgers A, Pan WH, Gu DF, Woodward M. Body mass index and cardiovascular disease in the Asia-Pacific Region: an overview of 33 cohorts involving 310 000 participants. Int J Epidemiol. 2004;33(4):751-8. Epub 2004/04/24. doi: 10.1093/ije/dyh163

dyh163 [pii]. PMID: 15105409.

30. Lewington S, Clarke R, Qizilbash N, Peto R, Collins R, Prospective Studies C. Age-specific relevance of usual blood pressure to vascular mortality: a meta-analysis of individual data for one million adults in 61 prospective studies. Lancet. 2002;360(9349):1903-13. PMID: 12493255.

31. Lewington S, Whitlock G, Clarke R, Sherliker P, Emberson J, Halsey J, et al. Blood cholesterol and vascular mortality by age, sex, and blood pressure: a meta-analysis of individual data from 61 prospective studies with 55,000 vascular deaths. Lancet. 2007;370(9602):1829-39. Epub 2007/12/07. doi: S0140-6736(07)61778-4 [pii]

10.1016/S0140-6736(07)61778-4 [doi]. PMID: 18061058.

32. Whitlock G, Lewington S, Sherliker P, Clarke R, Emberson J, Halsey J, et al. Body-mass index and cause-specific mortality in 900 000 adults: collaborative analyses of 57 prospective studies. Lancet. 2009;373(9669):1083-96. PMID: 19299006.

33. Di Angelantonio E, Sarwar N, Perry P, Kaptoge S, Ray KK, Thompson A, et al. Major lipids, apolipoproteins, and risk of vascular disease. JAMA : the journal of the American Medical Association. 2009;302(18):1993-2000. Epub 2009/11/12. doi: 10.1001/jama.2009.1619. PMID: 19903920; PMCID: PMCPMC3284229.

34. Singh GM, Danaei G, Farzadfar F, Stevens GA, Woodward M, Wormser D, et al. The age-specific quantitative effects of metabolic risk factors on cardiovascular diseases and diabetes: a pooled analysis. PloS one. 2013;8(7):e65174. Epub 2013/08/13. doi: 10.1371/journal.pone.0065174. PMID: 23935815; PMCID: PMCPMC3728292.

35. Lim SS, Vos T, Flaxman AD, Danaei G, Shibuya K, Adair-Rohani H, et al. A comparative risk assessment of burden of disease and injury attributable to 67 risk factors and risk factor clusters in 21 regions, 1990-2010: a systematic analysis for the Global Burden of Disease Study 2010. Lancet. 2012;380(9859):2224-60. Epub 2012/12/19. doi: 10.1016/S0140-6736(12)61766-8. PMID: 23245609; PMCID: PMCPMC4156511.

36. Danaei G, Ding EL, Mozaffarian D, Taylor B, Rehm J, Murray CJ, et al. The preventable causes of death in the United States: comparative risk assessment of dietary, lifestyle, and metabolic risk factors. PLoS medicine. 2009;6(4):e1000058. Epub 2009/04/29. doi: 10.1371/journal.pmed.1000058. PMID: 19399161; PMCID: PMCPMC2667673.

37. Hu FB, Rimm EB, Stampfer MJ, Ascherio A, Spiegelman D, Willett WC. Prospective study of major dietary patterns and risk of coronary heart disease in men. The American journal of clinical nutrition. 2000;72(4):912-21. Epub 2000/09/30. PMID: 11010931.

38. Fung TT, Willett WC, Stampfer MJ, Manson JE, Hu FB. Dietary patterns and the risk of coronary heart disease in women. Archives of internal medicine. 2001;161(15):1857-62. PMID: 11493127.

39. Fung TT, Rexrode KM, Mantzoros CS, Manson JE, Willett WC, Hu FB. Mediterranean diet and incidence of and mortality from coronary heart disease and stroke in women. Circulation. 2009;119(8):1093-100. Epub 2009/02/18. doi: 10.1161/circulationaha.108.816736. PMID: 19221219; PMCID: PMCPMC2724471.

40. Trichopoulou A, Bamia C, Trichopoulos D. Anatomy of health effects of Mediterranean diet: Greek EPIC prospective cohort study. BMJ (Clinical research ed). 2009;338:b2337. Epub 2009/06/25. doi: 10.1136/bmj.b2337. PMID: 19549997; PMCID: PMCPMC3272659.

41. Martinez-Gonzalez MA, de la Fuente-Arrillaga C, Lopez-Del-Burgo C, Vazquez-Ruiz Z, Benito S, Ruiz-Canela M. Low consumption of fruit and vegetables and risk of chronic disease: a review of the epidemiological evidence and temporal trends among Spanish graduates. Public health nutrition. 2011;14(12A):2309-15. Epub 2011/12/15. doi: 10.1017/s1368980011002564. PMID: 22166189.

42. Appel LJ, Sacks FM, Carey VJ, Obarzanek E, Swain JF, Miller ER, 3rd, et al. Effects of protein, monounsaturated fat, and carbohydrate intake on blood pressure and serum lipids: results of the OmniHeart randomized trial. JAMA : the journal of the American Medical Association. 2005;294(19):2455-64. PMID: 16287956.

43. Sacks FM, Svetkey LP, Vollmer WM, Appel LJ, Bray GA, Harsha D, et al. Effects on blood pressure of reduced dietary sodium and the Dietary Approaches to Stop Hypertension (DASH) diet. DASH-Sodium Collaborative Research Group. The New England journal of medicine. 2001;344(1):3-10. PMID: 11136953.

44. Appel LJ, Moore TJ, Obarzanek E, Vollmer WM, Svetkey LP, Sacks FM, et al. A clinical trial of the effects of dietary patterns on blood pressure. DASH Collaborative Research Group. The New England journal of medicine. 1997;336(16):1117-24. Epub 1997/04/17. doi: 10.1056/nejm199704173361601. PMID: 9099655.

45. He FJ, MacGregor GA. Effect of modest salt reduction on blood pressure: a meta-analysis of randomized trials. Implications for public health. Journal of human hypertension. 2002;16(11):761-70. Epub 2002/11/22. doi: 10.1038/sj.jhh.1001459. PMID: 12444537.

46. Estruch R, Ros E, Salas-Salvado J, Covas MI, Corella D, Aros F, et al. Primary prevention of cardiovascular disease with a Mediterranean diet. The New England journal of medicine. 2013;368(14):1279-90. Epub 2013/02/26. doi: 10.1056/NEJMoa1200303. PMID: 23432189.

47. Mozaffarian D. Mediterranean diet for primary prevention of cardiovascular disease. The New England journal of medicine. 2013;369(7):673-4. Epub 2013/08/16. doi: 10.1056/NEJMc1306659#SA3. PMID: 23944310.
